# Modification Mechanism of Multipolymer Granulated Modifiers and Their Effect on the Physical, Rheological, and Viscoelastic Properties of Bitumen

**DOI:** 10.3390/ma18174182

**Published:** 2025-09-05

**Authors:** Yao Li, Ke Chao, Qikai Li, Kefeng Bi, Yuanyuan Li, Dongliang Kuang, Gangping Jiang, Haowen Ji

**Affiliations:** 1 Engineering Research Center of Ministry of Education of Transportation Materials, Chang’an University, Xi’an 710064, China; cqlyo@163.com; 2 Tibet Autonomous Region High-Grade Highway Development and Emergency Support Center, Nyingchi Maintenance Center, Nyingchi 430074, China; 3 School of Civil Engineering and Architecture, Wuhan Institute of Technology, Wuhan 430074, China; 22404010089@stu.wit.edu.cn (K.C.); 22404010130@stu.wit.edu.cn (Q.L.); 22304010081@stu.wit.edu.cn (K.B.); GangpingJ@163.com (G.J.); jhw0616@outlook.com (H.J.); 4 School of Materials Science and Engineering, Chang’an University, Xi’an 710064, China

**Keywords:** granulated polymers, direct feed, modification mechanism, distribution state, adhesion

## Abstract

Polymer-modified bitumen is difficult to produce and often separates during storage and transport. In contrast, granular bitumen modifiers offer wide applicability, construction flexibility, and ease of transport and storage. This study involved preparing a multipolymer granulated bitumen modifier with a styrene–butadiene–styrene block copolymer, polyethylene, and aromatic oil. To elucidate the modification mechanism of a multipolymer granulated bitumen modifier on bitumen, the elemental composition of bitumen A and B, the micro-morphology of the modifiers, the changes in functional groups, and the distribution state of the polymers in the bitumen were investigated using an elemental analyzer, a scanning electron microscope, Fourier-transform infrared spectroscopy, and fluorescence microscopy. The effects of the multipolymer granulated bitumen modifier on the physical, rheological, and viscoelastic properties of two types of base bituminous binders were investigated at various dosages. The test results show that the Z_H/C_ ratio of base bitumen A is smaller than that of base bitumen B and that the cross-linking effect with the polymer is optimal. Therefore, the direct-feed modified asphalt of A performs better than the direct-feed modified asphalt of B under the same multipolymer granulated bitumen modifier content. The loose, porous surface structure of styrene–butadiene–styrene block copolymer promotes the adsorption of light components in bitumen, and the microstructure of the multipolymer granulated bitumen modifier is highly coherent. When the multipolymer granulated bitumen modifier content is 20%, the physical, rheological, and viscoelastic properties of the direct-feed modified asphalt of A/direct-feed modified asphalt of B and the commodity styrene–butadiene–styrene block copolymer are essentially identical. While the multipolymer granulated bitumen modifier did not significantly improve the performance of bitumen A/B at contents greater than 20%, the mass loss rate of the direct-feed modified asphalt of A to aggregate stabilized, and the adhesion effect reached stability. Image processing determined the optimum mixing temperature and time for multipolymer granulated bitumen modifier and aggregate to be 185–195 °C and 80–100 s, respectively, at which point the dispersion homogeneity of the multipolymer granulated bitumen modifier in the mixture was at its best. The dynamic stability, fracture energy, freeze–thaw splitting strength ratio, and immersion residual stability of bitumen mixtures were similar to those of commodity styrene–butadiene–styrene block copolymers with a 20% multipolymer granulated bitumen modifier mixing amount, which was equivalent to the wet method. The styrene–butadiene–styrene block copolymer bitumen mixture reached the same technical level.

## 1. Introduction

As the binder of the mixture, the performance of bitumen directly affects the road performance and service life of the bitumen pavement. The styrene–butadiene–styrene block copolymer (SBS) has been proven to significantly improve the high- and low-temperature performance, adhesion, resilience, and ductility of bitumen [1]. It is a commonly used modification additive. SBS absorbs the light components of bitumen, including saturated and aromatic fractions, through the intermediate segment of butadiene. This process of absorption results in the solubilization of the light components, leading to the formation of a network structure [2]. Experimental studies have shown that the styrene chain segments interact with the polar components in bitumen via intermolecular forces; subsequent adsorption of these polar components onto the nodes of the swollen network gives rise to the formation of stable physical cross-linking sites [3]. Simultaneously, the addition of a modest quantity of phase solvents and stabilizers during the designated preparation process and within the stipulated development conditions will ensure that SBS-modified bitumen exhibits optimal performance [4].

Scholars in various countries have carried out extensive research into the SBS-modified bitumen modification mechanism, enhancement effect, and preparation process. Lu [5] concluded that increasing the SBS content of modified bitumen decreases its stability and that star SBS-modified bitumen is less stable than linear SBS-modified bitumen at high temperatures. Zhang [6] prepared high-viscosity bitumen using SBS, a plasticizer, and a crosslinker; tested the effect of different modifiers on its low-temperature performance; and carried out infrared spectroscopy tests and differential thermal analysis tests on the prepared high-viscosity bitumen to study the modification mechanism and microstructure of different polymers in high-viscosity bitumen. Sheng [7] concluded that using a high-speed mixing method for desulfurization can reduce the agglomeration phenomenon of waste rubber powder particles, enhance the dissolution capacity, and improve the performance of waste rubber powder-modified bitumen. Shan [8] explored the linear and nonlinear properties of SBS-modified bitumen using Fourier-transform rheology and stress decomposition. The results show that the nonlinear characteristics of modified bitumen are more obvious than those of matrix bitumen and increase with the increase in SBS content, and the modified bitumen shows viscoelastic behavior under all the test conditions, and SBS can reduce the fluidity of modified bitumen and increase its elasticity ratio.

Unlike SBS’s flexible network, the reinforcement of the crystalline zones in plastic granules enhances high-temperature performance. Polyethylene (PE) is a polymer made from ethylene through the polymerization of linear polymers. It belongs to the class of long-chain aliphatic hydrocarbon polymers. As a bitumen modifier, PE can improve its performance, mainly because it can form a network-like structure that constrains the movement of bitumen molecules, effectively enhancing its viscoelasticity and reducing its sensory characteristics when heated [9]. KhanIM [10] used dynamic shear rheometer (DSR) tests to show that both low-density polyethylene (LDPE) and high-density polyethylene (HDPE) improve the rutting resistance and temperature sensitivity of bitumen. Goutham [11] obtained that waste plastics can be used as bitumen additives to increase the stability of bitumen mixtures, and a small amount of plastic can be used to replace some of the fibers by using spin compaction gauge (SCG) tests. Hu [12] modified bitumen with recycled packaging waste polyethylene (WPE) instead of common polymer modifiers and showed that WPE improved the high-temperature stability, low-temperature crack resistance, service life, and water stability of bitumen mixtures. Liang [13] investigated the behavior of the polyethylene phase in PE-modified bitumen by experimental and simulation methods. It was found that the reduction of polyethylene density and crystallinity resulted in easier migration of bitumen components into the intermolecular space, stronger interaction between the two, and higher solubility. Dalhat [14] found that for every 2% increase in PE, the performance grade (PG) increased by one grade. Habib [15] studied linear low-density polyethylene (LLDPE)- and HDPE-modified bitumen and found that thermoplastic copolymers significantly reduced base bitumen needle penetration, and the best modification effect was achieved when polyethylene blending was kept below 3 wt%. Wu [16] summarized the effect of recycled waste plastics such as HDPE, LDPE, polypropylene (PP), and polystyrene (PS) on bitumen and concluded that waste plastics improved the stiffness, rutting resistance, fatigue resistance of matrix bitumen mixtures, etc., but the compatibility between recycled plastics and bitumen was poor. Mahyar [17] utilized polyvinyl chloride (PVC) as an additive to improve the properties of bitumen and hot mix bitumen (HMA). The results showed that PVC improves the rheological properties of bitumen, and HMA specimens doped with PVC have better rutting resistance.

Currently, the most effective way to enhance the properties of bitumen is through the use of composite modification additives, which include thermoplastic elastomer materials, plastomer materials, and rubber-like materials. Fu [18] prepared SBS/waste rubber powder-modified bitumen by the chemical modification method and analyzed the viscoelastic properties of modified bitumen by rheometer test. The results indicated that the composite modification of SBS and waste rubber powder has certain improvements on the modified bitumen’s low-temperature and high-temperature performance. Zhang [19] used polyphosphoric acid and sulfur to improve the high-temperature performance and storage stability of SBS/SBR-modified bitumen. The test showed that polyphosphoric acid significantly improved the high-temperature performance of SBS/SBR-modified bitumen but reduced the storage stability. Ahmedzade [20] prepared modified bitumen using 40% HDPE, 25% ethylene propylene diene monomer (EPDM), and 17.5% tire rubber in mass fraction. The test results showed that the modified bitumen could increase the softening point, reduce the needle penetration, as well as improve the high-temperature performance, but the improvement for fatigue life was slightly worse compared to SBS-modified bitumen. Guo [21] used waste-reinforced polypropylene (RPP) and an SBS composite to prepare modified bitumen, and the test results showed that the composite modification of RPP and SBS further enhanced the cross-linking between the polymer and bitumen and significantly improved the rheological properties of bitumen. Ma [22] prepared recycled rubber–plastic- and SBS-modified binder blends. The results showed that the recycled rubber–plastic-modified bitumen could form a continuous phase of rubber and plastic, which improved the compatibility of the binder mixture and the properties of the bitumen mixture. Saeed [23] conducted rheological tests, Hamburg rutting tests, and dynamic creep tests and found that the high- and low-temperature properties and rutting resistance of the modified bitumen mixtures prepared by the addition of appropriate proportions of SBS and montmorillonite (MMT) were significantly improved. Zhe Hong [24] conducted indoor experimental studies, which verified that the incorporation of low-density polyethylene (LDPE) and ethylene–vinyl acetate copolymer (EVA) as modifiers can significantly enhance the permanent deformation resistance and elastic modulus of modified bitumen. COSTA [25] carried out a morphological structure analysis, storage stability test, and creep recovery test by adding EVA and HDPE to bitumen. The results showed that EVA-modified bitumen was similar to SBS-modified bitumen in terms of viscoelastic properties, but HDPE-modified bitumen was slightly higher than SBS-modified bitumen in terms of softening point and needle penetration. Mostafa [26] prepared (styrene–butadiene rubber) SBR-/PP-modified bitumen and evaluated its performance by bending beam fatigue, indirect tensile fatigue, and modulus of resilience tests. The results showed that SBR/PP can significantly improve the fatigue resistance and service life of modified bitumen mixtures.

The process of dry modified bitumen involves adding the modifier straight to the aggregate and then mixing the resulting bitumen mixture. This eliminates issues with wet modified bitumen storage stability and other issues, and it requires no large equipment and is suitable for on-site construction. Yucel [27] prepared dry warm mix rubber powder modified bitumen mixtures using different temperatures and compaction processes and investigated the effect of warm mixing agents on the internal structure and properties of dry rubberized bitumen mixtures. Shi [28] prepared a high-viscosity bitumen modifier, SPR, from SBS/C9 petroleum resin blends and investigated the chemical properties of SPR and its modification effect on bitumen. The results showed that SPR had a good modification effect on bitumen. Movilla-Quesada [29] used a dry process to prepare a polymer made by fusing waste polyethylene terephthalate (PET), HDPE, and PVC with aggregates at 180 °C, which was used to replace part of the bitumen, and then mixed with the bitumen to prepare bitumen mixtures. It was found that this polymer was stiffer than bitumen, thus improving the overall hardness of the mixture. Zhang [30] used a twin-screw extruder to prepare a PE-modified bitumen masterbatch, and the prepared masterbatch was pelletized and then blended into the base bitumen using the blending method to prepare polyethylene-modified bitumen. The results show that the polyethylene masterbatch prepared by a twin-screw extruder has better compatibility with bitumen and can significantly improve the softening point and reduce the degree of needle penetration. A large number of studies have shown that there is little difference in the road performance of wet and dry modified bitumen. Even when the content is the same, such as for water stability, the mixture prepared using the dry process performs better than that prepared using the wet process.

In recent years, researchers’ focus in the field of polymer-modified bitumen has had a certain bias: more focus has been placed on the impact of different types of polymer modifiers on bitumen performance and the formulation of various modifiers, while there is relatively less research on the modification mechanism between polymer particle modifiers and bitumen. At the same time, although there have been studies on the preparation of bitumen modifier using a variety of polymers, and the performance and process of polymer modifier have been discussed, the research on the modification mechanism of multi-component polymer particle modifiers and bitumen, its distribution law in the mixture, and process control methods is still relatively scarce, and the related issues have not been clearly explained.

In order to solve the above problems, this study used SBS, PE, aromatic oil, and other polymers to prepare a multi-component polymer particle bitumen modifier (MBM) and carried out a series of studies through the “macro micro” cross-scale research method. At the micro level, with the help of element analyzer, scanning electron microscope (SEM), Fourier-transform infrared spectrometer (FTIR), fluorescence microscope (FM), and other technical means, the element composition of matrix bitumen, the microstructure of modifier, the change of functional groups in modified bitumen and the distribution of a polymer in bitumen were deeply explored so as to reveal the modification mechanism between a multi-component polymer and bitumen. In terms of macro-performance research, the physical properties, rheological properties, and viscoelastic properties of DMA and DMB with different dosages were tested by three index tests: the dynamic shear rheological test, the 60 °C dynamic viscosity test, and the elastic recovery test. At the same time, the changes in adhesion between different types of aggregates and different amounts of modified bitumen were explored.

This study aims to achieve multiple goals: First, optimize the material composition of MBM and solve the defects of existing modifiers in storage and transportation. The second is to reveal the interaction mechanism between MBM and bitumen and its distribution in the mixture. The third is to clarify the optimal mixing parameters and mixing amount under the direct casting process. Fourth, verify the improvement effect of MBM on the pavement performance of the mixture and provide a cost-effective modification scheme for small projects and maintenance scenarios.

The research results not only provide a new choice for the selection of materials in road maintenance and repair but also provide a theoretical basis for the performance control of bitumen mixture under the direct casting technology and have important significance for the promotion and application of multi-component polymer particle bitumen modifiers in the road field.

## 2. Materials and Experimental Methods

### 2.1. Materials

Different types of modifiers and base bitumens exhibit varying modification effects on polymer-modified particles, and the selection of modifiers has a significant impact on the performance of modified bitumen. This chapter provides an explanation of base bitumen, SBS modifiers, and other polymer modifiers and introduces the experimental methods employed.

#### 2.1.1. Bitumen

The physical properties of two types of base bituminous binders (bitumen of A and B) are shown in Table 1.

#### 2.1.2. SBS Modifier

Styrene–butadiene–styrene block copolymer (SBS): As a thermoplastic elastomer, SBS improves the viscoelastic properties of bitumen by forming a three-dimensional network structure. The SBS selected in this study has a linear structure. The technical specifications of SBS are shown in Table 2.

#### 2.1.3. PE Modifier

PE molecular structure is irregular, showing a high degree of branching, a main chain with a large number of methyl branches and alkyl side chains, and even the formation of similar dendritic structures. The technical specifications of PE are listed in Table 3.

#### 2.1.4. Solubilizer

Solubilizers mainly contain polycyclic aromatic hydrocarbons, which can effectively promote the uniform dissolution of modifiers such as SBS in bitumen, thus forming stable blends in the polymer modification system [34,35]. The technical specifications of the solubilizer are listed in Table 4.

#### 2.1.5. CMB-SBS

The results of the performance indexes of commodity styrene–butadiene–styrene block copolymer (CMB-SBS)-modified bitumen used in the test are shown in Table 5. The testing methods mentioned in the table are all based on JTG E20-2011 [39].

#### 2.1.6. Aggregates

The aggregates used in the tests in this paper are limestone, and the technical specifications are shown in Table 6 and Table 7.

#### 2.1.7. Mineral Powder

The fillers used in the tests were all limestone mineral powder, and the indicators are shown in Table 8.

### 2.2. Experimental Methods

In this chapter, materials such as raw materials, modified particles, modified bitumen, and bitumen mixtures, along with their microscopic characteristics, macroscopic properties, and the corresponding testing methods, are introduced.

#### 2.2.1. Elemental Analysis

The base bitumen’s elemental composition was tested using the Vario EL cube CHNSO elemental analyzer (Elemental Analytical Systems, Frankfurt, Germany). The headquarters of the company is located in the Rhine Main region near Frankfurt, Germany. The elements C, H, O, N, and S in the bitumen samples were quantitatively converted into gaseous products such as CO_2_ and H_2_O by oxygen-enriched combustion at 950 °C and then quantitatively analyzed by a thermal conductivity detector after chromatographic separation.

#### 2.2.2. Scanning Electron Microscope (SEM)

In a scanning electron microscope, an electron beam emitted by an electron gun is focused to form a point light source, accelerated by an accelerating voltage to form a high-energy electron beam, and then focused into a micro-beam spot by a two-stage electromagnetic lens. Eventually, an end lens with a scanning coil causes the electron beam to bombard the surface of the sample in a raster scan pattern, exciting signals at different depths. These signals are captured by a probe receiver above the sample, amplified, and synchronized on a computer monitor for imaging.

#### 2.2.3. Fourier-Transform Infrared Spectroscopy (FTIR)

By using attenuated total reflection FTIR, the functional groups of the modifier were characterized to analyze their chemical compositions. The instrument is a Nicolet 6700 FTIR spectrometer manufactured by Thermo Fisher Scientific, headquartered in Waltham, Massachusetts, USA. The scanning frequency was 32 per minute, with wave numbers from 4000 cm^−1^ to 500 cm^−1^.

#### 2.2.4. Fluorescence Microscope Behavior Test (FM)

The M330F-3M830F fluorescence microscope from Shenzhen Aosvi Optical Instrument Co., LTD (Shenzhen, China) was used to capture images, which were then processed using Image View software. (The software version adopts the Image View image acquisition and processing system equipped with AOVSI’s research grade metallographic fluorescence microscope.) After 30 min of heating at 100 °C, the slides were allowed to cool at room temperature for two hours. The blue-violet monochromatic light source was at 420 nm, and the magnification ranged from 40 to 100 times.

#### 2.2.5. Technical Properties Tests

(1) Physical Properties

Tests were conducted for softening point ‘penetration’ ductility of bitumen according to tests T0604, T0605, and T0606 in JTG E20-2011.

(2) Rheological Properties

According to AASHTO T315, using the DHR-2 dynamic shear rheometer produced by TA company in the United States, the test temperature was 52–88 °C [40].

#### 2.2.6. Viscoelasticity Test

Regarding JTG E20-2011, the test was conducted using the T0620-2000 vacuum decompression capillary method [39]. The bitumen power viscosity of 60 °C was tested using the Changji company’s SYD-0620A bitumen power viscosity tester. The manufacturer of this instrument is Shanghai Changji Geological Instrument Co., Ltd. (Shanghai, China), located in Shanghai, China. The result of the test is the product of the viscosity tube constant and the flow time, as shown in Equation (1), where the specifications of the capillary viscometer and the dynamic viscosity range are shown in Table 9.

(1) η=K×t

where η is the dynamic viscosity of bitumen sample at measured temperature (Pa·s), K is the capillary calibration factor (Pa·s/s), and t is the liquid flow time (s)

#### 2.2.7. Elastic Recovery Test

The test was conducted using a digital extensometer at a test temperature of 25 °C, stretching the specimen at a speed of 50 mm/min, cutting the bitumen in the middle when the bitumen in the specimen was stretched to 100 mm, and then letting the bitumen specimen stand in water at 25 °C for 1 h to measure the change in length and calculate the elastic recovery rate in accordance with Equation (2).

(2) $$D=\frac{10-X}{10}\times 100$$

where D is the elastic recovery of the specimen (%), and X is the residual length of the specimen (cm).

#### 2.2.8. Adhesion Test

Adhesion test JTG E20-2011 in T 0616-1993 adhesion test of bitumen and coarse aggregate is used to check the adhesion between bitumen and coarse aggregate surface and to evaluate the water stripping resistance of coarse aggregate [39]. The visual method used to assess the stripping rate of bitumen from aggregate is subject to human factors and is prone to large deviations. Therefore, the adhesion of bitumen binder to aggregate was assessed by introducing a mass loss rate indicator.

Bitumen mixtures were tested using the rolled bottle method by sampling the bitumen mixtures through the quadratic method with a mixture mass of 50 ± 1 g, a rotation rate of 15 r/min, and a test temperature of 60 °C. After the roller bottle test, the bitumen binder peeled off the aggregate to varying degrees. The mass spalling rate is a measure of the change in the quality of the bitumen mixture, which in turn characterizes the degree of bitumen spalling. The mass loss rate, $R_{MS}$, of the bitumen mixture is calculated from Equation (3).

(3) $$R_{MS}=\frac{M_{c}-M_{s}}{M_{c}}\times 100\%$$

where $M_{c}$ is the mass of the bitumen mixture, and $M_{s}$ is the mass of the bitumen mixture after the test.

#### 2.2.9. Dispersion Uniformity

**Figure 1:**
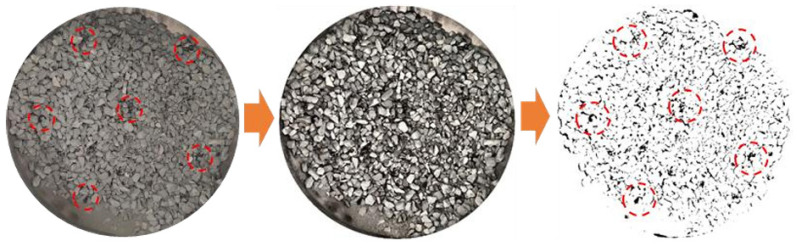
Image enhancement and binarization.

Image View software was used to enhance and binarize the images separately to observe the dispersion uniformity of MBM after mixing in the mixture. The obtained image is shown in Figure 1 and the red circle in the figure represents the distribution of MBM The experimental program design is shown in Table 10.

#### 2.2.10. High-Temperature Performance Test

The bitumen mixture specimens were prepared by the T0703 wheel rolling method in JTG E20-2011, and the rutting test was conducted by the T0719 [37]. We then evaluated the high-temperature stability of bitumen mixtures.

To evaluate the high-temperature performance of bitumen mixtures by the rutting test, high-viscosity modified emulsified bitumen mixtures were crushed into rutted specimens with a length, width, and height of 300 mm × 300 mm × 50 mm using a rutting instrument. Following the crushing process, the test specimen was placed in an oven set at a temperature of 60 °C for a duration of 60 min. Thereafter, the specimen was transferred to a chamber maintained at room temperature for 3 days, 7 days, 14 days, and 24 days. After completing these holding periods, the test specimen was inserted into the thermostat of the rutting instrument at a temperature of 60 °C for 5 h. Thereafter, the rutting instrument was activated, and the designated holding period was terminated. The instrument was then programmed to generate data automatically.

#### 2.2.11. Low-Temperature Performance Test

The fracture energy was used as the evaluation index of low-temperature performance to carry out the research on the low-temperature cracking performance of polymer-modified granular bituminous concrete. The loading apparatus was UTM-100, and the semicircular bending test (SCB) was used to test the low-temperature properties of bitumen mixtures. The depth of the open joint was a 5 mm gap, the insulation temperature was −10 °C, the insulation time was 4–6 h, and the loading rate of the UTM-100 test was 50 mm/min.

#### 2.2.12. Water Stability Test

The water stability of the mix was evaluated by using the T0709 water immersion Marshall test and the T0729 freeze–thaw splitting test in JTG E20-2011.

The bitumen mixture was compacted into a standard Marshall specimen with a diameter of 101 mm and a height of 63.5 mm (±1.3 mm) using a Marshall compactor. After 75 cycles of compacting, the specimen was placed in an oven at 60 °C for 60 min and then compacted a further 50 times.

For the Marshall immersion test, the specimens were immersed in water at 60 °C for 30 min and 48 h, respectively. They were then tested for Marshall stability, and the data were recorded to calculate the residual stability following immersion.

The freeze–thaw splitting test involves immersing the specimens in a water bath at 25 °C for 2 h. For the conditioning group, the specimens were first kept in water under vacuum, then frozen at −18 °C for 16 h, then placed in a water bath at 60 °C for 24 h, and finally placed in a water bath at 25 °C for 2 h. The specimens were then tested for splitting strength, and the data were recorded to calculate the freeze–thaw splitting ratio.

### 2.3. Preparation of MBM

MBM adopts the LJL-108 screw extruder for granulation molding. The manufacturer of the equipment is Changzhou Kaibang Drying Equipment Co., Ltd., located in Changzhou City, Jiangsu Province, China. The main parameters of the screw extruder are shown in Table 11.

**Figure 2:**
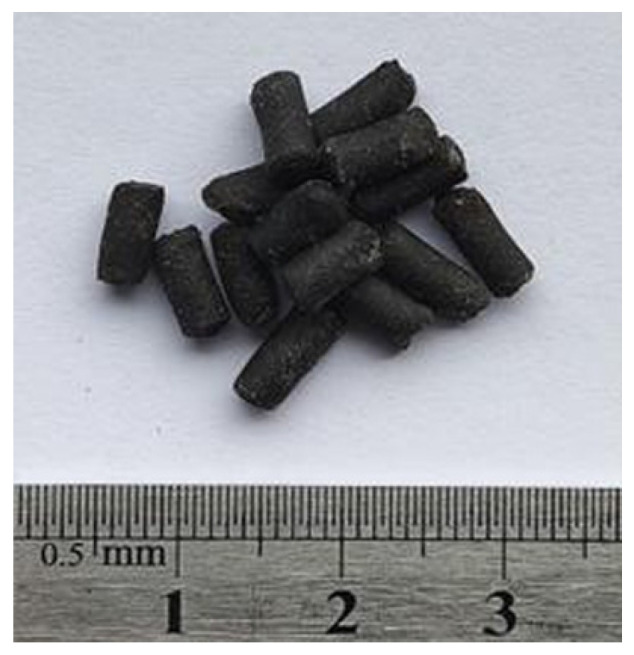
MBM.

This preparation process includes two stages: high-speed shearing and extrusion granulation. Stage 1: Firstly, the base bitumen was heated in an oven at 135 °C to melt it completely, then a weighed amount of solubilizer was added to the base bitumen, and the bitumen was subsequently heated to 180 °C. Add the corresponding mass of SBS modifier using 6000 r/min shear rate for shear so that the additives are uniformly dispersed in the bitumen to obtain the mixture A. Then, the corresponding mass of PE particles is added to the mixture A using 3000 r/min shear rate for shear so that the PE is uniformly dispersed to obtain the mixture B. In the second stage, the sheared finished mixture B was cooled to room temperature and put into the screw extruder for granulation to obtain MBM (as shown in Figure 2). The proportion of modifier composition in MBM is shown in Table 12.

### 2.4. Preparation of Modified Bitumen

To prepare the modified bitumen, the matrix bitumen was first heated in an oven at 135 °C to melt it completely, and the weighed MBM (in small quantities) was added to the matrix bitumen and heated to 180 °C. The modified bitumen was obtained by stirring at a rate of 500 r/min for 5 min. The categories of modified bitumen are shown in Table 13.

### 2.5. Technical Map

**Figure 3:**
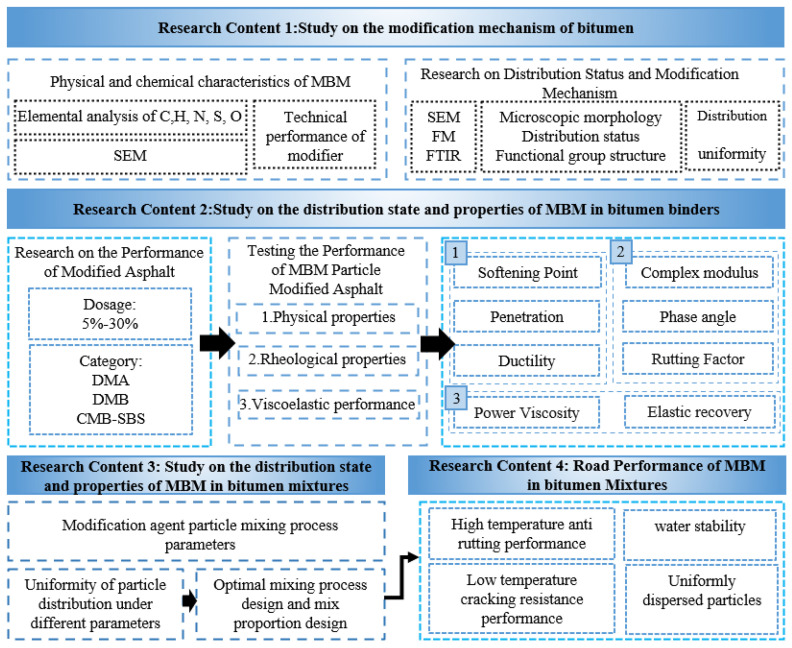
Technical map.

The technical map of this study is shown in Figure 3.

## 3. Results and Discussion

### 3.1. Study on the Modification Mechanism of Bitumen

#### 3.1.1. Elemental Analysis of Base Bitumen

The substance is composed of a large number of molecules, which in turn are made up of elements. The elemental composition of the substance plays a decisive role in its macroscopic properties. The different components of bitumen significantly affect its properties, especially when combined with different polymers [41]. Several studies [42,43,44,45,46,47] have confirmed that physical properties such as stiffness, elasticity, plasticity, adhesion, surface energy, and healing are influenced by the microstructure of bitumen. Among the four components of bitumen, aromatic hydrocarbons can dissolve the polymer at low concentrations, while the saturation fraction has a greater impact on modified bitumen. However, the bitumen content itself is less effective in dissolving the polymer [48].

**Figure 4:**
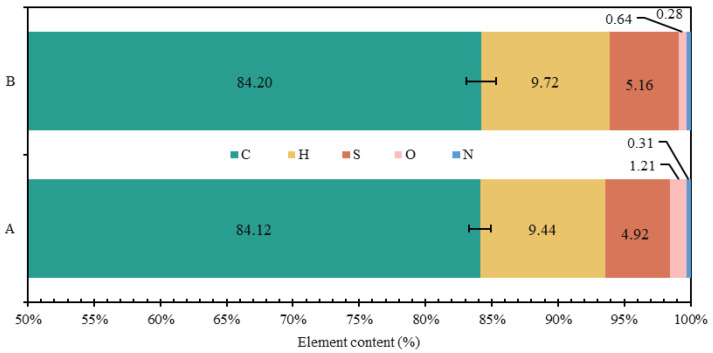
Element of base bitumen (A is base bitumen A, B is base bitumen B).

The composition of elements determines the macroscopic properties of a substance. Figure 4 and Table 14 display the elemental composition and element ratios of base bitumen A and base bitumen B. Oxygen content is calculated using the subtraction method. Figure 4 shows a sequential decrease in the levels of C, H, S, O, and N elements in the two base bitumens. C and H account for the largest proportions of S, O, and N, while the remaining heteroatoms make up a relatively smaller fraction. The hydrogen-to-carbon (Z_H/C_) ratio of base bitumen A and base bitumen B is 1.339 and 1.378, respectively. There is a significant difference in the percentage of heteroatoms such as S, O, and N between base bitumen A and B, which are 95.3%, 189.1%, and 110.7%, respectively, while the percentage difference between C and H is relatively small, at 99.9% and 99.7%, respectively.

The smaller the Z_H/C_ ratio, the fewer saturated hydrocarbons are present in the bitumen, and the more cyclic and aromatic ring structures it contains. The Z_H/C_ ratio of base bitumen A is smaller than that of base bitumen B, indicating that base bitumen A contains more cyclic and aromatic ring structures. The aromatic content in bitumen plays an essential role in the compatibility between bitumen and polymer; the higher the aromatic content, the better the performance of modified bitumen [42]. Therefore, base bitumen A is more effective in cross-linking the polymer with bitumen and promoting the solubilization and dispersion of the polymer within the bitumen.

#### 3.1.2. Microscopic Morphology Analysis of Modifier and MBM

**Figure 5:**
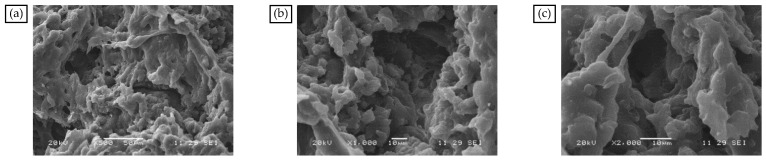
SEM images of modifiers: (**a**) SBS at 50×, (**b**) SBS at 500×, and (**c**) SBS at 1000×.

The SBS modification mechanism of bitumen is mainly a physical modification. When the polymer forms small particles dispersed within the bitumen, microscopic polymer particles in the bitumen, by the external temperature, load, and other factors, provide a resistance to deformation in bitumen so that the bitumen has the macro-performance of low-temperature performance enhancement and load-resistant capacity [49]. The SEM images of SBS are shown in Figure 5. As can be seen from Figure 5, the surface of the SBS modifier is loose and porous, which can effectively enhance the contact area between the SBS modifier and bitumen and has a good promotion effect on the adsorption of light components in bitumen.

**Figure 6:**
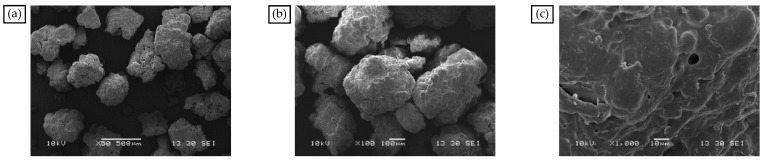
SEM images of modifiers: (**a**) PE at 50×, (**b**) PE at 100×, and (**c**) PE at 1000×.

From Figure 6a–c, the PE particles are 280–740 μm, irregular, and spherical. PE is a crystalline polymer. When the temperature rises to the melting point of PE, the internal molecular rotation and vibration intensification caused by the structural regularity of the destruction occur in PE molecules, so that the transformation of the amorphous structure can be dissolved and dispersed in bitumen under high-speed mixing [50].

**Figure 7:**
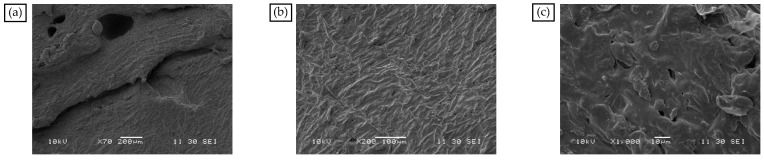
SEM images of MBM: (**a**) MBM at 70×, (**b**) MBM at 200×, and (**c**) MBM at 1000×.

In the presence of aromatic oil, SBS and PE cause the MBM surface to completely dissolve and disperse to create a mesh structure, as shown in Figure 7a–c. SBS and PE can absorb some of the lightweight components and continuously dissolve them when the magnification is increased to 1000 times. The surface pits and groove-like phenomena are more evenly distributed, and the overall distribution is uniform. PE and SBS are compatible with the base bitumen and are uniformly dispersed in the base bitumen under mechanical movement. SBS surface polymer chain segments can diffuse into the bitumen during high-speed mixing. Therefore, when MBM is mixed with base bitumen and aggregates at high temperatures, MBM can quickly dissolve in the base bitumen and adhere to the aggregate surface.

#### 3.1.3. FTIR Analysis of Modifier and MBM

**Figure 8:**
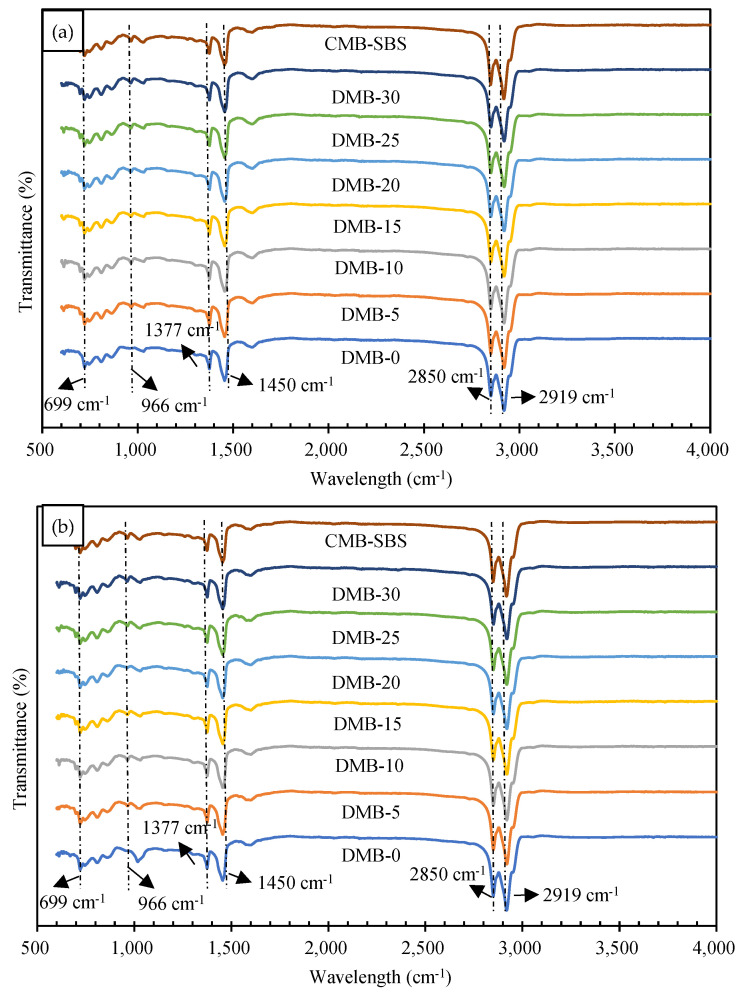
FTIR spectrum of DMA and DMB: (**a**) DMA; (**b**) DMB.

The infrared spectral test results of two base bitumens, modified bitumen and CMB-SBS, are shown in Figure 8. Figure 8a shows the results of base bitumen A and different contents of DMA and CMB-SBS tests, and Figure 8b shows the results of base bitumen B and different contents of DMB and CMB-SBS tests.

As can be seen from the figure, the more pronounced absorption peaks present in the IR spectrum are 699 cm^−1^, 966 cm^−1^, 1377 cm^−1^, 1450 cm^−1^, 1600 cm^−1^, 2850 cm^−1^, and 2915 cm^−1^ in that order. Compared with base bitumen, 699 cm^−1^ and 966 cm^−1^ appeared in DMA, DMB, and CMB-SBS as the characteristic absorption peaks of SBS, and no other new characteristic peaks appeared, which only showed that the area size of some characteristic peaks varied; therefore, MBM and base bitumen were mainly physically mixed.

**Figure 9:**
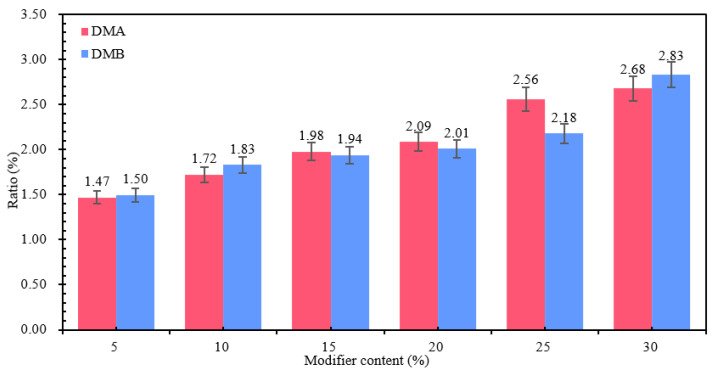
The ratio of area to total area at the peaks of 699 cm^−1^ and 966 cm^−1^.

The absorption peak of the trans-vibration of the C=C double bond of butadiene in SBS is 966 cm^−1^, while the absorption peak of the monosubstitution of the styrene ring in SBS is 699 cm^−1^. The ratio of the area at the two peaks to the total area is shown in Figure 9. From Figure 9, it can be seen that the ratios of 699 cm^−1^ and 966 cm^−1^ in DMA and DMB gradually become larger with the increase in MBM doping. This is mainly because the proportion of the SBS modifier in MBM increases with the increase in MBM doping, and the degree of dissolution of the SBS modifier and base bitumen increases.

The ratio of the sum of the areas at 699 cm^−1^, 966 cm^−1^, 1377 cm^−1^, 1450 cm^−1^, 1600 cm^−1^, 2915 cm^−1^, and 2850 cm^−1^ peaks in DMA and DMB at different doping levels is shown in Table 15. As can be seen from Table 15, the ratios at 1377 cm^−1^, 1450 cm^−1^, 2915 cm^−1^, and 2850 cm^−1^ have not changed significantly. While at 1600 cm^−1^ peak, its area share has an increasing trend with the increase in doping amount, while its ratio DMA > DMB.

The characteristic peak at 1600 cm^−1^ is the result of the stretching vibration of the hydroxyl group C=O of aldehydes, ketones, and acids and the double bond C=C of the benzene ring skeleton, mainly with the presence of aromatic hydrocarbons. Therefore, the content of aromatic oils in modified bitumen increases with the increase in MBM admixture of modified particles, and the area share of DMA at 1600 cm^−1^ is larger than the share of DMB, mainly due to the different contents of elements in the base bitumen A and B. From Section 3.1.1, the smaller the value of the hydrogen-to-carbon atom ratio Z_H/C_, the more aromatic ring structure in the chemical composition of the bitumen. The hydrogen-to-carbon atom ratio of base bitumen A is smaller than that of base bitumen B, indicating that base bitumen A contains more cyclic and aromatic ring structures. Therefore, the aromatic hydrocarbon content of DMA is greater than that of DMB.

#### 3.1.4. MBM Distribution in Bitumen

**Figure 10:**
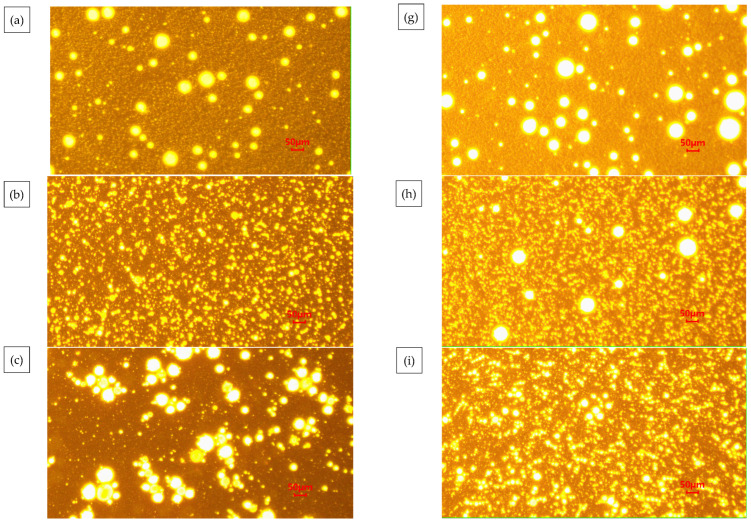

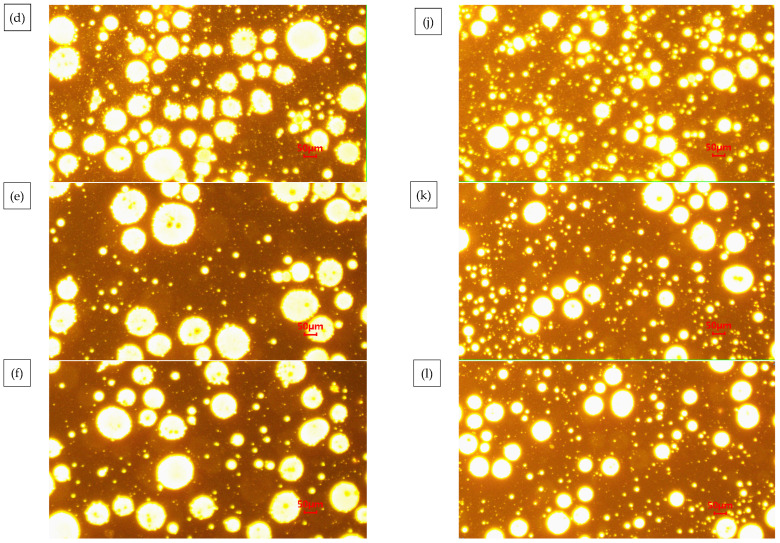
Distribution state of MBM in bitumen: (**a**) 5% DMA; (**b**) 10% DMA; (**c**) 15% DMA; (**d**) 20% DMA; (**e**) 25% DMA; (**f**) 30% DMA; (**g**) 5% DMB; (**h**) 10% DMB; (**i**) 15% DMB; (**j**) 20% DMB; (**k**) 25% DMB; (**l**) 30% DMB.

**Figure 11:**
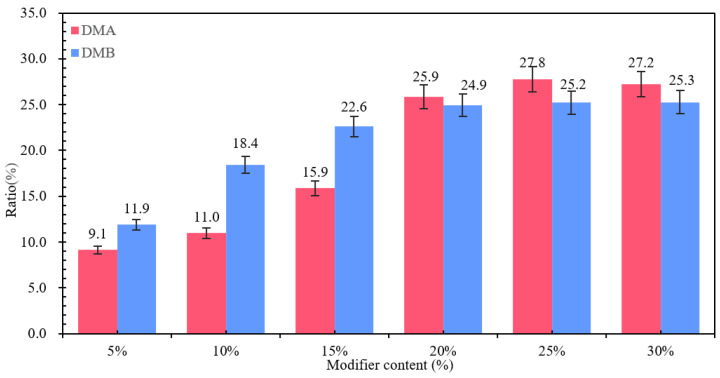
Particle area proportion of DMA and DMB in fluorescent images.

The dispersion state of the polymer in the bitumen reflects the compatibility of the polymer with the bitumen and, therefore, can directly reflect the modification effect of the modifier on the bitumen on a microscopic scale. The fluorescence images of different MBM doping are shown in Figure 10, and the percentage of particle area in different MBM doping is shown in Figure 11.

As can be seen in Figure 10, the number of particles and the particle size of the polymer in the MBM keep changing as the MBM doping increases. When the MBM doping was in the range of 5–15%, the polymer particles in the fluorescence image were mainly monodisperse, with uniform spatial distribution and large particle spacing, and the average spacing was around 3–5 times the particle diameter, and the density of the polymer particles increased linearly with the concentration, but the overall loose structure was still maintained. When the MBM doping is 20%, the distribution state is transformed, the particle density increases significantly, and a small-scale cluster structure is formed in some areas; when the MBM doping is in the range of 25–30%, a significant aggregation phenomenon occurs, and the particles form a continuous-phase structure, but the overall distribution is still relatively uniform. This is mainly due to the fact that the polymer, when blended with bitumen, absorbs the lighter components of the bitumen and then dissolves, forming a two-phase structure in which the dissolved polymer phase coexists with the bitumen phase [51]. PE belongs to the crystalline polymers. After melting PE from the crystalline state into an amorphous state, bitumen is absorbed in the lightweight components of the swelling, with the original folded polyethylene molecular chain unfolding, and then, through the high-speed mixing of the shearer, it is evenly distributed in the bitumen [52].

As can be seen from Figure 11, the area share of the particles increases approximately linearly with the increase in MBM, but levels off after the MBM reaches 20%. At low content (5–15%), the particles are more dispersed, while at high content (20–25%), the particle size becomes larger due to increased inter-particle interactions, so the area share of the particles increases, but at 30%, the area share of the particles decreases. It is mainly due to the fact that after high-speed shear, the polymers, such as SBS and PE, are gradually subdivided into shorter chain segments, and the activity of the molecular chain is enhanced with the shortening of the length, which promotes the absorption and dissolution of bitumen oil by the polymers, such as SBS. The polymer in the bitumen increases with increasing MBM content, so the area share of the particles increases with increasing content in the range of 5% to 25%. While at 30% dosing, the viscoelastic properties of bitumen limit the dispersion of particles, the continuous-phase structure of bitumen is disrupted, the particles cannot be uniformly dispersed, and some of the particles may settle or migrate to the interface due to gravity, resulting in a decrease in the effective dispersion area.

#### 3.1.5. MBM Distribution in Bitumen Mixtures

**Figure 12:**
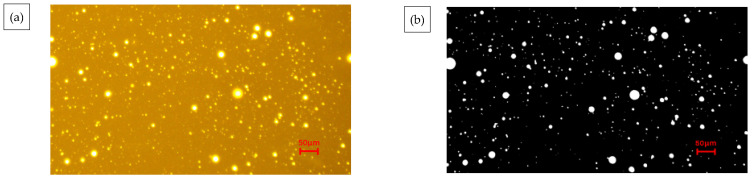

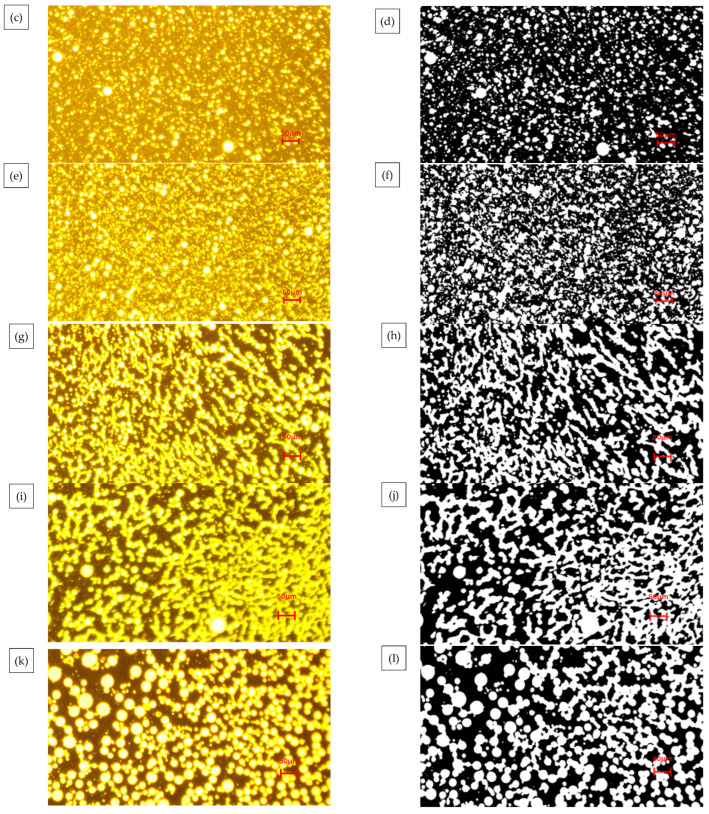
Fluorescence diagrams of different types of MBM in bitumen mixtures: (**a**,**b**) 5% of MBM; (**c**,**d**) 10% of MBM; (**e**,**f**) 15% of MBM; (**g**,**h**) 20% of MBM; (**i**,**j**) 25% of MBM; (**k**,**l**) 30% MBM.

The fluorescence images of different contents of MBM in bitumen mixture and the percentage of particles in bitumen mixture with different contents of MBM are shown in Figure 12 respectively. As can be seen from Figure 12, the polymer particles are uniformly distributed in the bitumen mixture, with no significant aggregation phenomenon, the particle boundaries are clear, and the interfacial compatibility with bitumen is better, which can form a stable multiphase system. The fluorescence images of MBM doping in the range of 5% to 15% showed a small size and good dispersion of polymer particles. After 10% MBM doping, the density of particle distribution in the fluorescence image increased significantly with the increase in MBM doping, and after 20% MBM doping, the size of particles became larger, and a continuous phase was formed in some regions. Compared with the distribution state fluorescence image of MBM in bitumen binder, MBM is more uniformly distributed in the bitumen mixture, and the particle size is smaller, which is mainly due to the high-temperature conditions of the bitumen mixture mixing process. Mechanical mixing under the action of aggregate can effectively increase the dispersibility of MBM so that MBM is uniformly dispersed in the bitumen and aggregates to improve the adhesion between bitumen and aggregates and thus enhance the modified bitumen mixture road performance.

**Figure 13:**
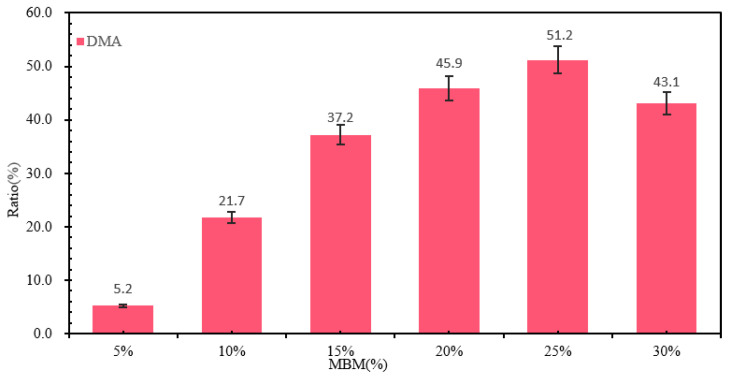
Percentage of particles in bitumen mixtures with various MBM content.

From Figure 13, it can be seen that the percentage of MBM’s particle area in bitumen binder in the fluorescence image shows a trend of first increasing and then decreasing with the increase in content. It is consistent with the trend of MBM in bitumen binders in Section 3.1.4. However, except for the 5% content, the proportion of MBM particles in bitumen mixtures is larger than that in binder, which increases by 3.8, 7.3, 9.2, 10.8, and 8.8 times, respectively, compared with that of the 5% content. This is mainly because the polymer in MBM is better dispersed uniformly in the bitumen mixture under mechanical mixing and high temperature.

### 3.2. Study on the Distribution State and Properties of MBM in Bitumen Binders

#### 3.2.1. Physical Properties of Modified Bitumen

(1) Softening Point

**Figure 14:**
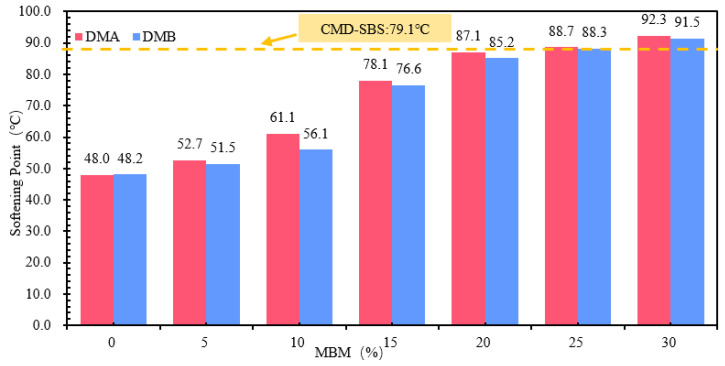
Softening point of modified bitumen.

The softening point test results of modified bitumen with different MBM content are shown in Figure 14, which shows that the softening point is positively correlated with the MBM content, and the softening point of DMA-30 and DMB-30 is improved by 92.3% and 89.8%, respectively, compared with that of the base bitumen. The softening point of the modified bitumen with an MBM content of 15% is not much different from that of the modified bitumen with CMA-SBS, and it is greater than that of CMA-SBS-modified bitumen with an MBM content of 20%.

Solubilizer in the aromatic oil component of its molecular structure and bitumen gum, similar to the bitumen colloid system, can effectively integrate into the bitumen colloid system, effectively promote SBS and other modifiers, and dissolve the bitumen uniformly in the polymer-modified particles to form a stable blending system. Under the mechanical action of high-speed shear, PE particles uniformly dispersed in bitumen can play a bridge role, so that the bitumen colloidal particles through the PE particles are connected, so that the bitumen from the sol–gel-type to the gel-type colloidal structure of the transformation significantly enhances the bitumen visco-elastic qualities so that the high-temperature performance of bitumen has improved significantly [53].

(2) Penetration

**Figure 15:**
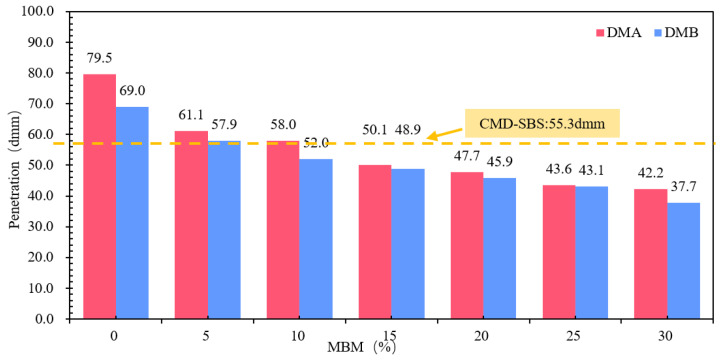
Penetration of modified bitumen.

Different MBM contents of modified bitumen penetration test results are shown in Figure 15. Figure 15 shows that, with the increase in MBM content, the modified bitumen needle penetration was gradually reduced. MBM content from 0% to 30% DMA-30 and DMB-30 needle penetration compared to the base bitumen was reduced by 46.9% and 45.4%, respectively. Penetration decreases gradually with MBM content, and the softening point increases continuously with MBM content. This is mainly due to the addition of MBM, SBS, and PE. MBM will absorb the light component of the base bitumen, resulting in the reduction of the light component and bitumen to form a continuous phase [53], so that the modified bitumen thermal stability and the molecular mobility of bitumen are reduced, resulting in the bitumen hardening and viscosity significantly increasing. The physical properties are manifested in the softening point increasing and needle penetration decreasing [54,55].

(3) Ductility

**Figure 16:**
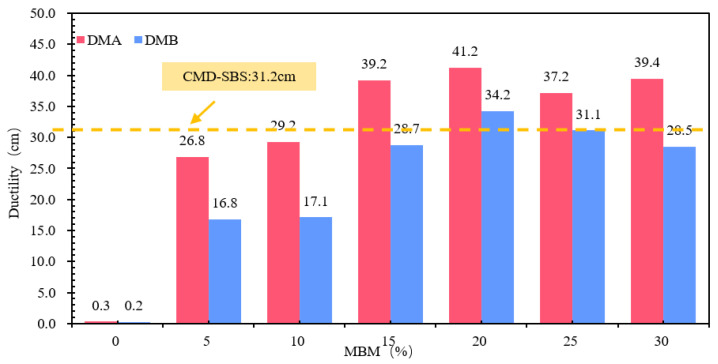
Ductility of modified bitumen.

The results of different MBM content-modified bitumen ductility tests are shown in Figure 16. Compared to the base bitumen, DMA-30 and DMB-30 ductility were increased 130 times and 141 times, and DMA-5 and DMB-5 were increased by 47.0% and 69.6%. DMA and DMB ductility with MBM content showed a tendency to increase and then decrease, with a content of above 15% DMA and DMB. The duration of DMA and DMB with more than 15% content is more than 25 cm, of which DMA is more than 30 cm. The difference between DMA-10-, DMB-15-, and CMA-SBS-modified bitumen ductility is not significant. This is mainly due to the plasticizing effect of aromatic oils in the solubilizer, which can reduce the glass transition temperature (Tg) of the bitumen and give the bitumen better low-temperature ductility. At the same time, with the MBM doping increases, SBS is fully dissolved and dispersed under the action of aromatic oils, resulting in more SBS from the segmented distribution into a continuous distribution, so that the ductility of the modified bitumen increases. On the other hand, with the increase in MBM doping, PE content in modified bitumen gradually increased, so that the bitumen becomes more brittle at low temperatures, especially when subjected to tensile stress, and on the modified bitumen surface, some of the internal inhomogeneous part of the surface of the bitumen produced fine cavities, which gradually developed into microfine grain, and the molecular chain fracture occurred at low-temperature stretching [56], so the modified bitumen ductility shows a first increase after the decreasing trend.

The differences in softening point and 5 °C ductility between base bitumen A and B were small, and the differences in needle penetration were large. In the range of 5–30% MBM content, the same content of DMA penetration, softening point, and ductility test results are greater than the DMB, indicating that the modifier in the base bitumen A has a greater effect than the base bitumen B due to the different types of bitumen, making the modifier in the bitumen dissolve and disperse in different degrees, thus making the difference in physical properties between DMA and DMB.

In order to further improve the reliability of data trends, a univariate ANOVA (α = 0.05) was performed to obtain a linear trend fit. The experimental data on the physical properties of modified bitumen are shown in Table 16.

Softening point: The slope is approximately +1.65 °C per 1% MBM, with an R^2^ value of 0.94. The quadratic term shows a minor effect (R^2^ = 0.96), indicating that the softening point increases monotonically and significantly with the admixture content.

Penetration: The slope is approximately −1.12 dmm per 1% MBM, with an R^2^ value of 0.87. The quadratic fitting yields an R^2^ of 0.97, suggesting an overall significant decrease, with a more gradual decline observed at higher admixture contents.

Ductility: A significant quadratic trend is observed (R^2^ = 0.92), characterized by an initial increase followed by a subsequent decrease with increasing admixture content. The maximum ductility occurs within the range of 15–20%, which aligns with the statement in the text that “15–20% is optimal”.

#### 3.2.2. Complex Modulus and Phase Angle of Modified Bitumen

**Figure 17:**
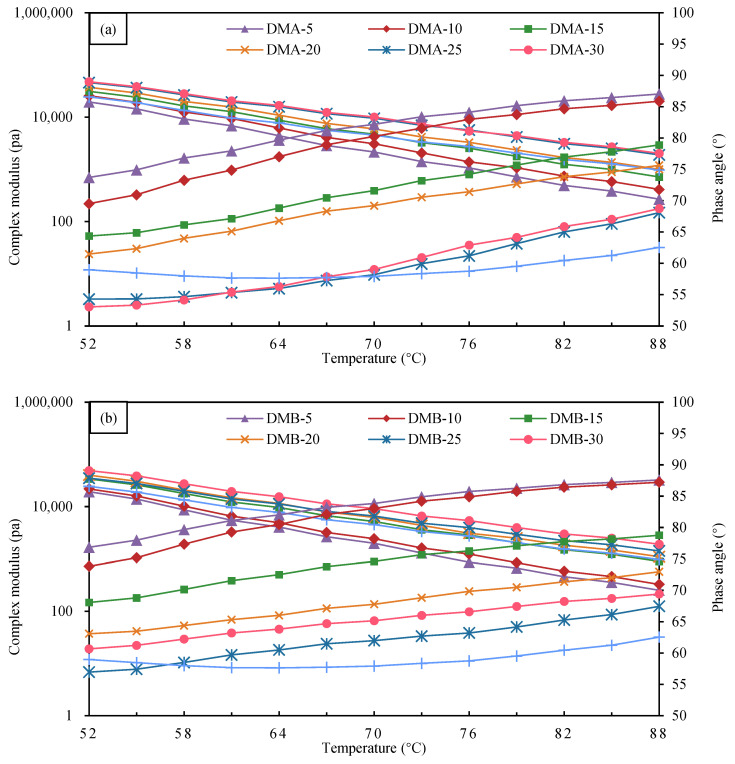
The complex shear modulus and phase angle of DMA/DMB with different modifiers: (**a**) DMA; (**b**) DMB.

The composite modulus and phase angle test results of DMA, DMB-modified bitumen, and CMB-SBS with different particle blends are shown in Figure 17, where Figure 17 shows the composite modulus and phase angle test results of DMA-modified bitumen and CMB-SBS with 5–30% particle blends, and Figure 10b shows the composite modulus and phase angle test results of DMB-modified bitumen and CMB-SBS with 5–30% particle content.

According to Figure 17, the composite modulus of DMA- and DMB-modified bitumen progressively rises with increasing particle admixture at the same temperature, while gradually decreasing with rising temperature in the range of 5 to 30% particle admixture. When granule blending was increased from 5% to 30% at 88 °C, the composite modulus of DMA-modified bitumen increased by 53.8%, 165.8%, 273.4%, 606.4%, and 652.6%, respectively; for DMB-modified bitumen, the composite modulus increased by 29.8%, 256.3%, 256.3%, 336.8%, 462.2%, and 656.0%, respectively. It demonstrates how increasing the modifier content in modified bitumen can improve the material’s resistance to deformation [57].

In the range of 5–30% particle content, the phase angle of both DMA- and DMB-modified bitumen increased to different degrees with the increase in temperature, while for MBM, the phase angle of DMA- and DMB-modified bitumen decreased to different degrees with the increase in particle content at the same temperature. For DMA-modified bitumen, the phase angle decreased by 1.2°, 8.1°, 11.4°, 18.9°, and 18.3° at 88 °C when the particle blending was increased from 5% to 30%. For DMB-modified bitumen, the phase angle decreased by 0.4°, 8.8°, 14.6°, 18.2°, and 20.1°. It is shown that the addition of MBM increases the elastic properties and decreases the viscous properties of bitumen, and the elastic properties become stronger and the viscous properties become weaker with the increase in particle dosing [10]. It is mainly due to the adsorption of the modifier and bitumen in the particles: when forming a stable three-dimensional network structure, the adhesion between bitumen molecules is enhanced, thus limiting the transformation of bitumen to a viscous flow state [11,58,59].

The sensitivity of MBM in base bitumen A was lower than that of base bitumen B, as evidenced by the fact that the composite modulus and phase angle of DMA-modified bitumen changed less with the change of particle content at 88 °C than did DMB-modified bitumen. Nonetheless, DMA-modified bitumen outperformed DMB-modified bitumen in terms of shear and deformation resistance at the same content, suggesting that MBM improved the rheological characteristics of the modified base bitumen A.

#### 3.2.3. Rutting Factor of Modified Bitumen

Rutting factor can characterize the high-temperature rutting resistance of bitumen binders; the larger the rutting factor, the stronger the rutting resistance of bitumen binders at high temperatures [60].

**Figure 18:**
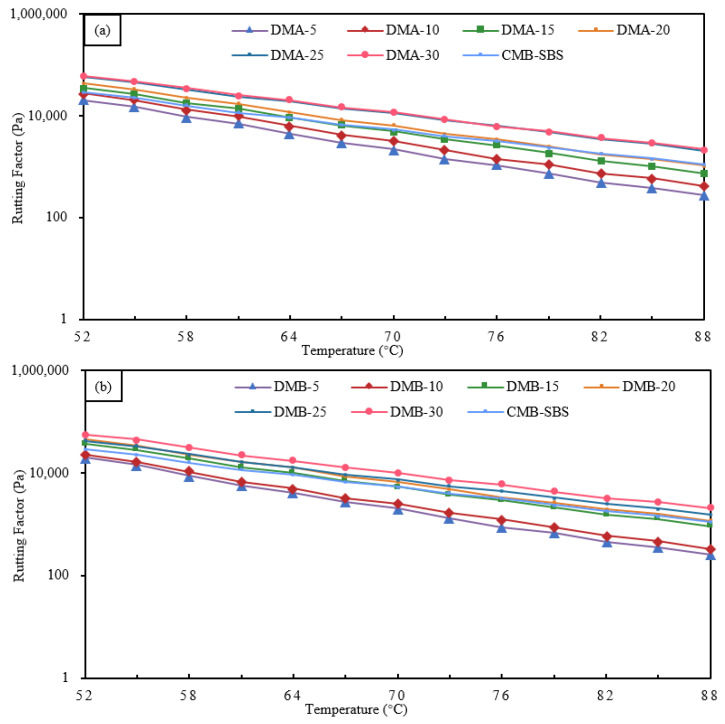
The rutting factor of DMA/DMB with different modifiers: (**a**) DMA; (**b**) DMB.

The rutting factor test results of DMA- and DMB-modified bitumen and CMB-SBS with different particle blends are shown in Figure 18. In the range of 5 to 30% granule blending, the rutting factor of DMA- and DMB-modified bitumen gradually decreases with increasing temperature, and it gradually increases with increasing granule blending at the same temperature. For DMA-modified bitumen, increasing the granule blending from 5% to 30% at 88 °C increased the composite modulus by 54.0%, 170.5%, 285.0%, 660.4%, and 706.4%, respectively; for DMB-modified bitumen, increasing the granule blending from 5% to 30% at 88 °C increased the composite modulus by 29.9%, 262.9%, 262.9%, 356.4%, 706.8%, and 508.1%, respectively. The phase angle change will be influenced by changes in the ratio of viscous to elastic components of the bitumen binder; the more viscous the bitumen binder, the bigger the phase angle, the lower the rutting factor, and the poorer the rutting resistance [1,61]. At the same temperature, as particle mixing rises, the phase angle of DMA- and DMB-modified bitumen gradually increases, as does the rutting factor. The modified bitumen’s viscous and elastic properties gradually deteriorated and improved, respectively, as a result of increased particle mixing, modifier particles, and bitumen adsorption, which limited the bitumen to a viscous flow state transformation. As a result, the rutting factor and rutting resistance of modified bitumen improved [62].

There is a significant correlation between the rheological properties and microstructure of DMA, DMB with different dosages: in the low dosage stage of 5%~15%, MBM particles are monodisperse with large spacing (about 3–5 times the particle diameter) and only slightly restrict the flow of bitumen molecules through swelling, resulting in a gradual increase in complex modulus, a slow decrease in phase angle, and limited improvement in rutting factor. When the dosage reaches 20%, the particle density significantly increases and forms a small-scale cluster structure, initially constructing an interwoven “particle bitumen” network. At this point, the complex modulus jumped significantly, while the phase angle significantly decreased, and the rutting factor exceeded 10^4^ Pa, reaching the level of wet SBS-modified bitumen technology. In the high dosage range of 25% to 30%, particles form a continuous dense network, and bitumen transforms into a dispersed phase. The complex modulus reaches its peak, while the phase angle is reduced to its lowest point, with elastic dominant material deformation and rutting factor exceeding 10^5^ Pa. In addition, DMA has a lower hydrogen-to-carbon ratio, higher aromatic content, more uniform particle dispersion, and better rheological properties than DMB at the same dosage.

#### 3.2.4. Dynamic Viscosity of Modified Bitumen

**Figure 19:**
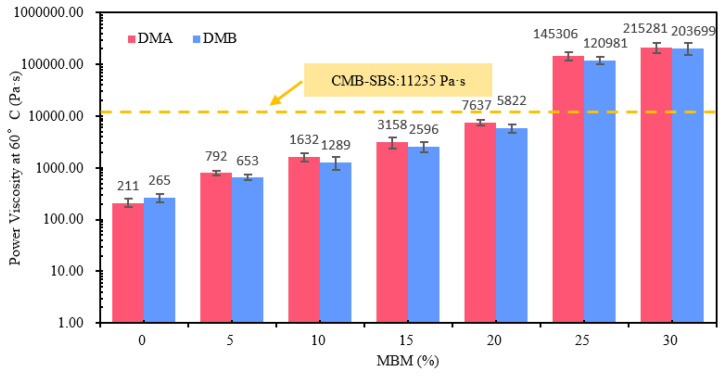
Dynamic viscosity of DMA- and DMB-modified bitumen with different contents at 60 °C.

The dynamic viscosity of DMA and DMB at 60 °C with varying doses is shown in Figure 19. As can be observed from Figure 19, the dynamic viscosity increased by 1.5, 3.9, 8.8, 21.0, 455.5, and 767.7 times over the base bitumen, while the 60 °C dynamic viscosity of DMA increased by 2.8, 6.7, 14.0, 35.2, 687.7, and 1019.4 times. The 60 °C dynamic viscosity of modified bitumen showed a typical three-stage nonlinear growth with increasing MBM content. At 5% dosing, the modifier in MBM is dispersed in the continuous phase of bitumen as isolated particles, and the viscosity increase is gentle; in the range of 5% to 20%, MBM forms localized clusters through interfacial interactions, and the viscosity increase is accelerated. With 20% MBM as the viscosity sudden increase inflection point, at this time, the viscosity grows over 35 times and 21 times compared to the base bitumen, indicating that the network in the modified bitumen forms a critical concentration. When the MBM content is in the range of 20–30%, the modifier in MBM forms a continuous network skeleton, and the bitumen becomes a dispersed phase. Mainly due to the MBM increase, SBS in modified bitumen continues to increase, and in the high-temperature mechanical mixing, the SBS modifier uniformly dispersed in the bitumen and dissolution development, so that the bitumen component changes from the sol–gel type to the direction of the gel type, with SBS further increasing the degree to which gelation deepens, and SBS particles see an increase in the interactions between each other and the effect of the force, and the bitumen components change from sol–gel type to gel type direction, with a further increase in the degree of gelatinization continuing to deepen, and SBS particles in the interaction and the force increase. With the formation of a dense, continuous mesh structure, the modified bitumen network pocket effect is enhanced [63,64,65], and viscosity increases. It is mainly due to the fact that with the increase in MBM, the SBS in the modified bitumen continues to increase, and under the mechanical mixing at high temperatures, the SBS modifier is uniformly dispersed in the bitumen and dissolved and developed, which makes the bitumen components change, and the bitumen is transformed from the sol–gel type to the gel direction, and with the further increase in SBS, the degree of gelatinization does not, and with the promotional effect of the solubilizing agent and high temperature, the bitumen components diffuse in the PE phase, so that the PE surface molecules dissolve, forming an interfacial transition layer between PE and bitumen, enhancing the interaction between PE and bitumen [66] and hindering the movement of bitumen molecules, effectively improving the viscosity of bitumen.

#### 3.2.5. Elastic Recovery

**Figure 20:**
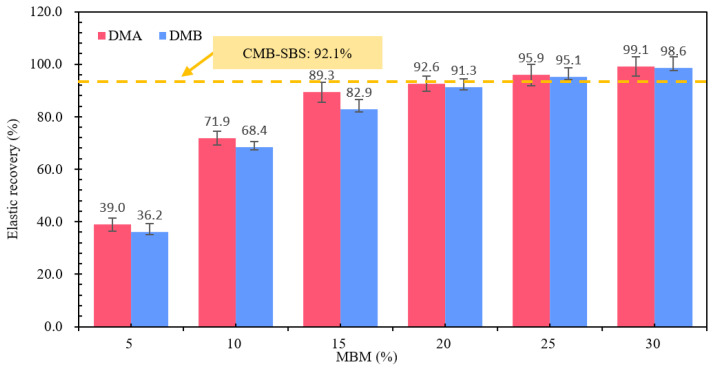
Elastic recovery rate for DMA and DMB.

The variation in the elastic recovery rate of different contents of DMA/DMB is shown in Figure 20. From Figure 20, it can be seen that the elastic recovery rate of both types of modified bitumen, DMA/DMB, is positively correlated with the MBM content. DMA resilience recovery rates increased by 32.9%, 17.4%, 3.3%, 3.3%, and 3.2%, respectively, and DMB resilience recovery rates increased by 32.2%, 14.5%, 8.4%, 3.8%, and 3.5%, respectively. Modified bitumen with an MBM content of 5% to 15% showed a consistent change in the modified bitumen elastic recovery rate change law. The MBM content of 20% to 30% is primarily caused by SBS, which is a component of the material’s polymer chain structure and is added to the bitumen to be adsorbed in the light component. The volume of expansion and viscoelasticity can be improved in the bitumen after the formation of a spatial network following the dissolution of the elasticity, when the modified bitumen’s elastic recovery rate shows a tendency to flatten characteristics. With the increase in content, the network structure becomes more perfect, and after reaching a certain content, the change of its performance will tend to level off. With the increase in the network structure, it becomes more perfect, and after reaching a certain amount, its performance changes tend to level off. When MBM is around 20%, the elastic recovery rate of DMA and DMB tends to be the same as that of CMB-SBS.

### 3.3. Study on the Distribution State and Properties of MBM in Bitumen Mixtures

#### 3.3.1. Distribution State of MBM in the Bitumen Mixtures

**Figure 21:**
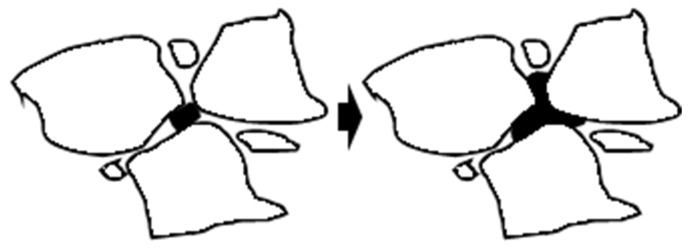
Melting process of modifier particles.

The direct-feed modifier is put into the mixing pot and mixed dry with the aggregate first, then sprayed into the bitumen for wet mixing. In terms of physical phenomena, the process can be broken down into three sub-processes: melting, viscous flow, and melt mixing [67]. As shown in Figure 21.

**Figure 22:**
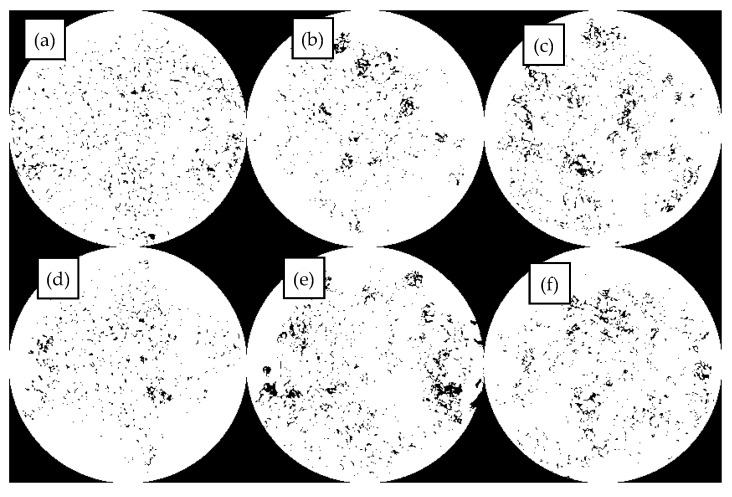
Distribution of MBM in bitumen mixtures at different mixing temperatures: (**a**) 20% mixing temperature at 170 °C; (**b**) 20% mixing temperature at 190 °C; (**c**) 20% mixing temperature at 210 °C; (**d**) 30% mixing temperature at 170 °C; (**e**) 30% mixing temperature at 190 °C; (**f**) 30% mixing temperature at 210 °C.

**Figure 23:**
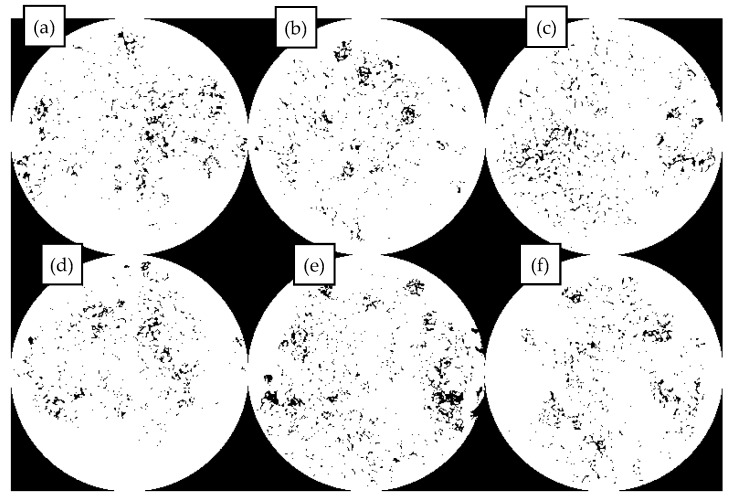
Distribution of MBM in bitumen mixtures with different mixing times: (**a**) 20 per cent mixing time of 60 s; (**b**) 20 per cent mixing time of 90 s; (**c**) 20 per cent mixing time of 120 s; (**d**) 30 per cent mixing time of 60 s; (**e**) 30 per cent mixing time of 90 s; (**f**) 30 per cent mixing time of 120 s.

**Figure 24:**
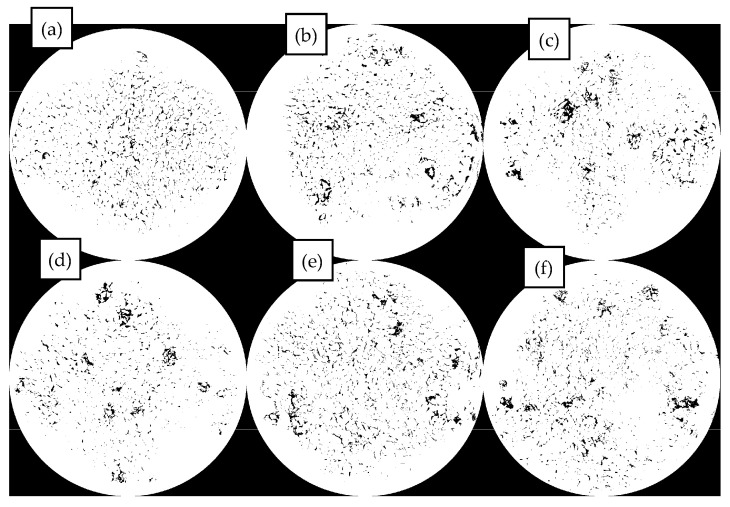
Distribution of MBM in bitumen mixtures with different contents: (**a**) 5 per cent content; (**b**) 10 per cent content; (**c**) 15 per cent content; (**d**) 20 per cent content; (**e**) 25 per cent content; (**f**) 30 per cent content.

This section systematically studies the distribution characteristics and pattern of MBM in the mixture system with the variation of mixing temperature, mixing time, and dosage. Refer to Figure 22, Figure 23, and Figure 24, respectively.

In Figure 22, under different mixing temperatures, the dispersion of MBM in bituminous mixtures becomes more uniform as the temperature increases. At a 20% dosage, elevated temperatures intensify the formation of “clusters.” At 170 °C, MBM with 20% and 30% dosages can disperse uniformly without obvious “clusters.” For the 30% dosage, when the temperature rises from 190 °C to 210 °C, the number of “clusters” decreases; however, excessively high temperatures will damage the interface stability due to the volatilization of light components. Therefore, the optimal mixing temperature is determined to be 185–195 °C.

In Figure 23 under different mixing durations, MBM with a 30% dosage shows poor dispersibility at 60 s, while that with a 20% dosage exhibits better dispersibility. Extending the mixing duration to 90 s can improve dispersion uniformity; further extending it to 120 s reduces agglomeration but causes segregation between coarse and fine aggregates. Thus, the optimal mixing duration is approximately 90 s.

In Figure 24 under the optimal conditions of 190 °C and 90 s, MBM with a 5% dosage disperses uniformly without clustering. “Clusters” begin to appear at a 10% dosage, which is related to the melting of MBM at high temperatures and its adhesion to aggregates. At a 30% dosage, the degree of clustering is lower than that at 25%, possibly due to the overlap of MBM affecting surface detection. Adding base bitumen can increase the contact area between MBM and bitumen, promote the integration and dispersion of polymers, and thereby enhance the performance of the mixtures.

#### 3.3.2. Mixing Ratio Design

(1) Gradation design

**Figure 25:**
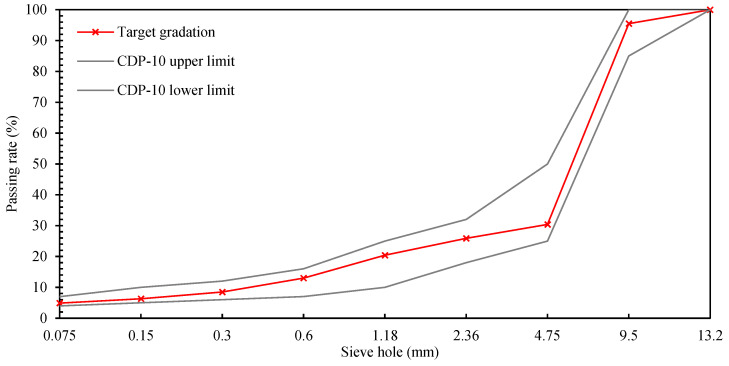
CDP-10 bitumen mixture production ratio grading curve.

After trial mixing, the proportion of mineral aggregate in CDP-10 bitumen mixture is: 5–10 mm:3–5 mm:0–3 mm:mineral powder = 62:10:23:5. Bitumen mixture synthetic grading is shown in Table 17, and the grading curve is shown in Figure 25.

(2) Optimum bitumen content

**Figure 26:**
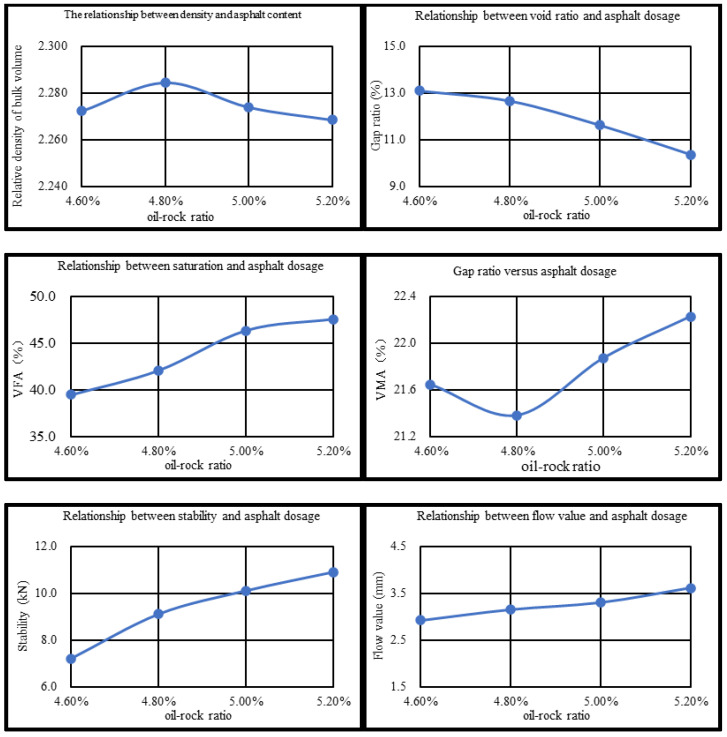
Marshall specimen parameters of CDP-10 mix with different oil/stone ratios.

Marshall tests were carried out on CDP-10 with oil/stone ratios of 4.6%, 4.8%, 5.0%, and 5.2%, respectively, with specimens compacted at temperatures ranging from 170 °C to 175 °C and 50 blows on each side. The correspondence between gross bulk relative density, void ratio, stability, flow value, voids filled with bitumen (VFA), and voids in mineral aggregate (VMA) and oil–rock ratio is shown in Figure 26. As can be seen from Figure 26, the relative density of gross volume shows the trend of increasing and then decreasing with the increase in oil/stone ratio, which is mainly due to the fact that when the oil/stone ratio is low, the bitumen in the mix is not enough to sufficiently coat the aggregates, and the internal pores are more, so its density is lower. At the appropriate oil/stone ratio, the bitumen gradually fills the aggregate skeleton voids, and the density increases. When the oil–rock ratio is too large, excessive bitumen in the mixture will form a lubrication layer, leading to an increase in the spacing of aggregate particles, structural expansion, and a decrease in density. The mixture void fraction decreased by 3.3%, 3.7%, and 4.6% with the increase in the oil-to-rock ratio. This is due to the increase in bitumen content, which increases the mutual adhesion between aggregate and bitumen and reduces the internal voids in the mix. Mix stability, size, and oil/stone ratio are positively correlated with the trend; this is due to the increase in bitumen content, the aggregates between the bonding force increase, and the bonding force and internal friction synergistic effect, which leads to mix stability enhancement [68].

The saturation of the mix does not meet the requirements when the oil/gravel ratio is above 5.0%, and its stability is less than 8 kN when the oil/gravel ratio is 4.6%. When the CDP-10 oil–rock ratio is 4.8%, all the technical indexes of its Marshall test meet the specification requirements. Therefore, the optimum oil/stone ratio for CDP-10 was determined to be 4.8%, and the results of the optimum oil/stone ratio Marshall test are detailed in Table 18.

(3) The Schellenberg Bitumen Leakage Test

The Schellenberg bitumen leakage test evaluates the amount of bitumen binding material that precipitates out of the bitumen mixture at elevated temperatures and drains off the excess free bitumen, thus evaluating the maximum amount of bitumen that can be used in a bitumen mixture. We carried out a leakage test on modified bitumen mix CDP-10 at a test temperature of 185 °C. When the oil–rock ratio is 4.8%, the average precipitation loss rate of CDP-10 is 0.12%, which meets the requirement of the performance index that the precipitation loss rate of the binding material should not be more than 0.3%. The results of the leakage analysis test are shown in Table 19.

(4) The Fort Kentucky Flyaway Test

The Fort Kentucky flyaway test can be used to assess the extent of dispersion due to bitumen content or insufficient bonding in the event of dislodgement of aggregates from the pavement surface under traffic loading. The results of the mixture dispersion test are shown in Table 20. When the oil–rock ratio is 4.8%, the average dispersion loss rate of the CDP-10 mixture is 8.0%, which meets the requirement of the performance index that the dispersion loss rate of the combined material is not more than 10%.

#### 3.3.3. Aggregate–Bitumen Adhesion Properties

(1) Aggregate and bitumen spalling condition

**Figure 27:**
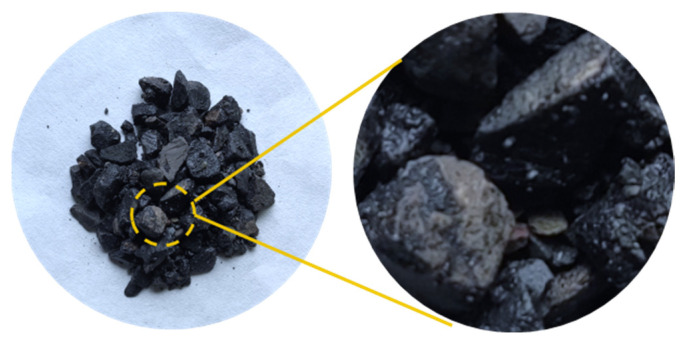
Matrix bitumen mixture surface shedding condition.

**Figure 28:**
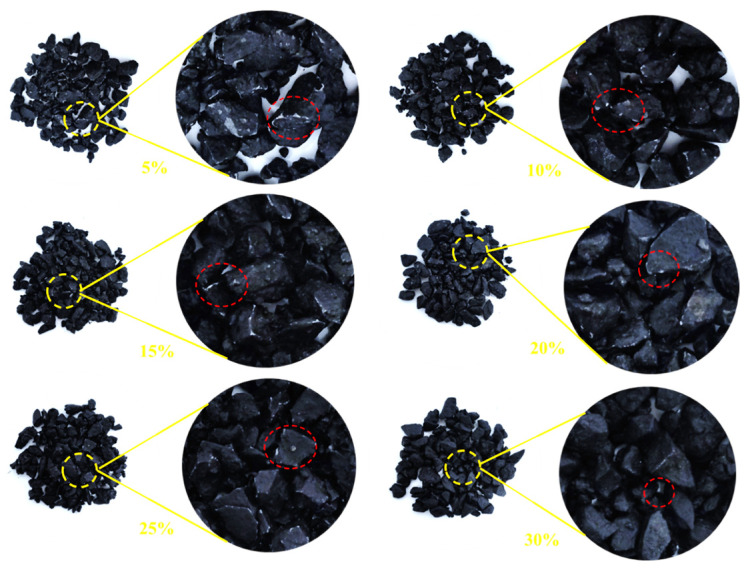
The surface shedding state of DMA bitumen mixtures with different contents.

The interface between bitumen and aggregate mastic is the weakest place of the bitumen mixture, which will directly affect its structural strength, water stability, fatigue, and other major properties, thus restricting the service life of bitumen pavement. The stronger the cohesion of the modified bitumen, the better the adhesion of the bitumen to the aggregate, and the better it resists water damage [69]. The image of the surface spalling state of base bitumen mixtures is shown in Figure 27, and the image of the surface spalling state of DMA bitumen mixtures with different contents is shown in Figure 28.

As can be seen in Figure 27, the bitumen in the bitumen mixture has spalled off extensively, indicating that the matrix bitumen has weak adhesion to the bitumen.

As can be seen from Figure 28, there was no significant stripping of DMA from the aggregate, and only some of the bitumen spalled off at the corners of the aggregate in the range of MBM content of 5% to 15%, while the spalling phenomenon gradually diminished with the increase in MBM content, and at 30%, no significant spalling could be seen. It shows that the addition of MBM can effectively enhance the adhesion properties of bitumen to aggregates. This is mainly due to the fact that with the increase in MBM dosing, the increase in polymer content, such as SBS and PE in MBM, effectively improves the viscosity of bitumen, resulting in enhanced adhesion between bitumen and aggregate. Therefore, the modified bitumen–aggregate adhesion property is enhanced with the increase in MBM content.

(2) Rate of loss of bitumen mixture quality

**Figure 29:**
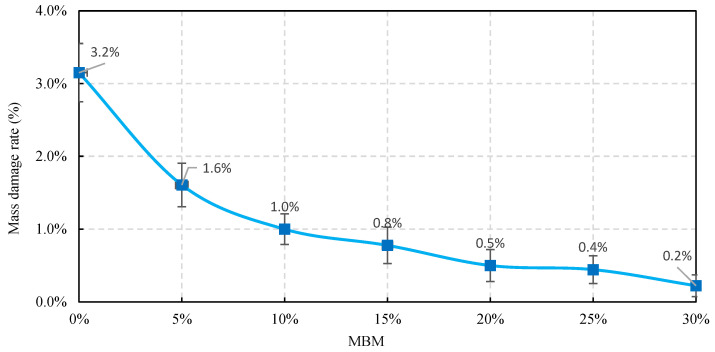
Bitumen mixture mass loss rate.

The change in mix quality before and after the test is shown in Table 21, and the rate of loss of bitumen mix quality at different test times is shown in Figure 29. As can be seen from Table 21 and Figure 29, the post-test mass loss rate of bitumen mixtures decreases gradually as the MBM content increases. However, compared to the base bitumen, the mass damage rate of the DMA bitumen mixture was reduced by 47.9%, 67.6%, 74.8%, 83.8%, 85.6%, and 92.8%, respectively, indicating that the incorporation of MBM can significantly improve the adhesion properties between bitumen and aggregates.

### 3.4. Road Performance of Bitumen Mixtures

#### 3.4.1. High-Temperature Performance

**Figure 30:**
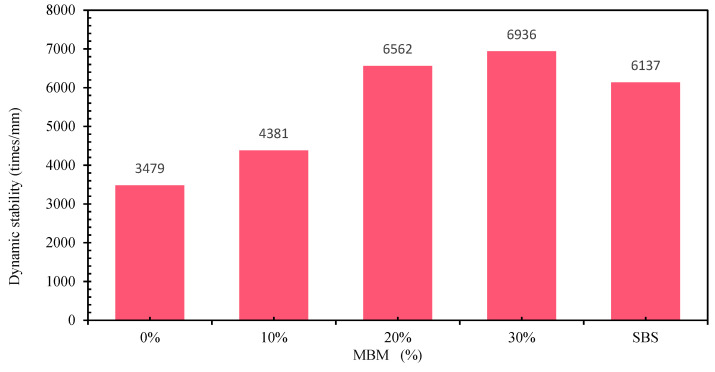
Dynamic stability of different types of bitumen mixtures.

With high-temperature conditions or long-term loading, bitumen pavement will produce significant deformation. Deformation in the part that cannot be restored is permanent deformation; rutting is the most common and most dangerous disease of bitumen pavement. The rutting test, as the most commonly used test method for evaluating the high-temperature stability of bitumen mixtures in China’s standard specifications, has the advantages of being simple, convenient, easy to operate, has good correlation, and has become a reliable test method for evaluating high-temperature performance indexes [70]. The results of the dynamic stability tests with different contents of DMA, CMB-SBS, and matrix bitumen mixtures are shown in Figure 22. MBM can effectively improve the rutting resistance of bitumen mixtures at high temperatures, as shown in Figure 30, where the dynamic stability of DMA bitumen mixtures increased by 22.9%, 88.6%, and 99.4%, respectively, when compared to matrix bitumen mixtures as the MBM content was increased. This is primarily because MBM’s polymer is evenly distributed throughout the bitumen mixture during mechanical mixing and high temperatures, improving aggregate and bitumen adhesion and the bitumen mixture’s high-temperature performance. At 20% MBM content, the mix’s dynamic stability has surpassed that of the CMB-SBS mix in comparison.

#### 3.4.2. Low-Temperature Performance

**Figure 31:**
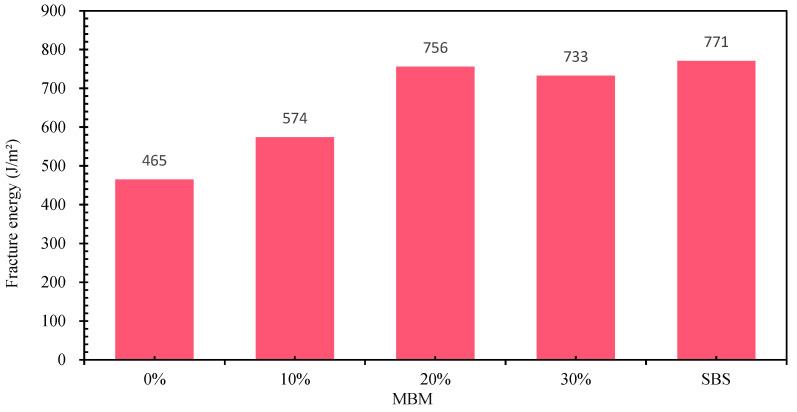
Fracture energy of different types of bitumen mixtures.

The low-temperature cracking resistance of bitumen mixtures is significantly affected by the low-temperature properties of bitumen, and its low-temperature cracking resistance is key to road service life and safety. In this study, the SCB test was used to evaluate the low-temperature performance of DMA bitumen mixtures with different contents. The SCB test results are shown in Figure 31, from which it can be seen that the fracture energies of DMA, CMB-SBS, and base bitumen mixtures with different contents are 574 J/m^2^, 756 J/m^2^, 733 J/m^2^, 771 J/m^2^, and 465 J/m^2^, respectively, and the fracture energies of DMA bitumen mixtures are improved by 23.2%, 62.6%, and 57.6%, respectively. This indicates that MBM can effectively improve the low-temperature cracking resistance of bitumen mixtures. It is mainly due to the continuous-phase system formed by intertwining the polymer and bitumen in MBM, which is an effective reinforcement to the structure of the bitumen mixture. Moreover, the polymer in MBM is uniformly dispersed in the bitumen mixture under high temperatures and mechanical mixing and forms a continuous-phase system with bitumen that is not easy to deform in a coordinated manner, which enhances the cohesion of the bitumen mixture and strengthens the adhesion between bitumen and aggregates, thus improving the low-temperature deformation resistance of the bitumen mixture.

#### 3.4.3. Water Stability

Bitumen pavement, in the service process, will be subject to temperature, water, vehicle loads, and other external conditions together, making the bitumen mixture pavement subject to continuous erosion from rainwater, the surface of the aggregate loose spalling, and other phenomena. Bitumen mixture in the repeated action of the vehicle load, rainwater into the bitumen mixture inside the gap will produce dynamic water pressure; water will gradually penetrate the bitumen and aggregate in the middle of the aggregate by the joint action of external forces, and water is prone to spalling. In this study, a freeze–thaw split test and an immersion Marshall test were used to evaluate the water stability properties of DMA, CMB-SBS, and matrix bitumen mixtures.

**Figure 32:**
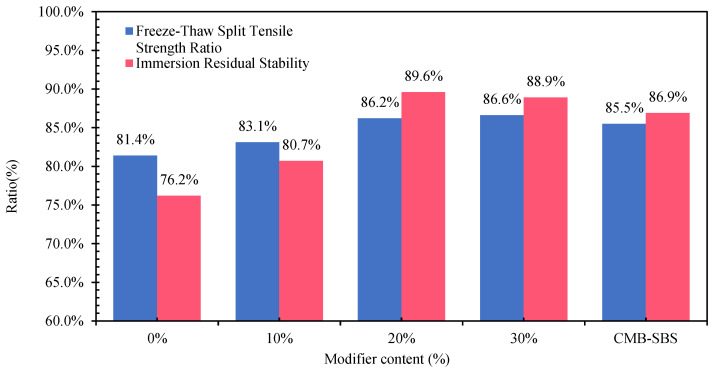
Freeze–thaw splitting test and water immersion Marshall for different types of bitumen mixtures.

The results of the freeze–thaw splitting test and immersion Marshall test for different contents of DMA, CMB-SBS, and matrix bitumen mixtures are shown in Figure 32. As can be seen from Figure 24, with the increase in MBM dosing, the freeze–thaw splitting strength of CDP-10 mixes increased by 2.1%, 5.9%, and 6.4%, respectively, compared with that of the matrix bitumen mixtures, and with the increase in MBM dosing, the immersion residual stability of CDP-10 mixes increased by 5.9%, 17.6%, and 16.7%, respectively, compared with that of the matrix bitumen mixtures. It shows that MBM can effectively improve the water loss resistance of bitumen mixtures. Compared with CMB-SBS, the freeze–thaw split strength ratio and the residual stability in water immersion were greater than those of the CMB-SBS bitumen mixture at a 20% MBM content, indicating that the MBM content can reach the level of the wet SBS-modified bitumen mixture at about 15%.

## 4. Conclusions

In this study, a multipolymer granulated bitumen modifier was developed using SBS, PE, solubilizers, and other auxiliary materials. To address the limitations of traditional wet-process modified bitumen production—specifically its poor adaptability to small-scale projects and high tendency to cause material waste—MBM employs a dry direct feeding process. During construction, materials can be dosed on demand, effectively avoiding material surplus and energy waste. This process simplifies operational procedures, enhances transportation and storage convenience, and significantly improves construction flexibility and cost-effectiveness. Meanwhile, optimized mixing process parameters were determined to ensure uniform dispersion of MBM across different dosage levels, compatibility with on-site mixing equipment, reduced reliance on large-scale production facilities, and effectively improved economic efficiency. In this study, a series of analytical techniques were employed to characterize the system: an elemental analyzer was used to determine the elemental composition of A/B base bitumen, scanning electron microscopy (SEM) and fluorescence microscopy (FM) were utilized to observe the micro-morphology of SBS/PE/MBM mixtures, and Fourier-transform infrared spectroscopy (FTIR) was applied to investigate changes in functional groups between the modifier and bitumen. These analyses collectively elucidated the distribution state of MBM in bitumen and the underlying modification mechanism. The effect of MBM doping at concentrations ranging from 0% to 30% on A and B type bitumen was evaluated through a suite of tests, including physical property measurements, dynamic shear rheology tests, dynamic viscosity tests, and elastic recovery tests. Image binarization technology was employed to observe the dispersion homogeneity of MBM in mixtures under varying temperature conditions and mixing times, facilitating an assessment of the adhesion effect between MBM-doped mixtures (0–30% dosage) and aggregates. Additionally, water-soaked Marshall tests, freeze–thaw splitting tests, and rutting tests were conducted to compare the road performance of mixtures with different MBM contents against those containing CMB-SBS. The conclusions are as follows:

(1) The ratio of hydrogen and carbon atoms in base bitumen A is smaller than in base bitumen B, which is better cross-linked with polymer bitumen. Therefore, the DMA’s physical, rheological, and viscoelastic properties are better than those of the DMB. The SBS surface is loose and porous, which plays a good role in promoting the adsorption of light components in bitumen and the formation of a stable structure in bitumen solubility. The MBM surface, along with the PE, plays a role in the aromatic oils becoming fully soluble and dispersed to form a reticular structure;
(2) The changes in the peak area share of DMA and DMB at 699 cm^−1^ and 966 cm^−1^ increased with an increase in the MBM content (0–30%). The change in peak area share at 1600 cm^−1^ for DMA and DMB, however, was mainly related to different aromatic oil content and base bitumen fractions. The density and size of MBM particles in bitumen and bitumen mixtures were positively correlated with content, with the best adhesion to aggregates being achieved at a content of 20%;
(3) Increasing the MBM doping from 5% to 30% resulted in a decrease in the needle penetration and an increase in the softening point of DMA, while the opposite trend was observed for DMB. The needle penetration of DMA-30 decreased, and the softening point increased compared to CMB-SBS. The elongation of DMA and DMB increased initially and then decreased with MBM doping, reaching a peak at 20% doping. This was 32% and 9.6% higher than that of CMB-SBS, respectively. Kinetic viscosity and elastic recovery at 60 °C exhibited a three-stage nonlinear increase, with the viscoelastic properties of DMA and DMB reaching the same level as CMB-SBS at an MBM content of 15–20%;
(4) Image binarization was used to determine the optimum mixing temperature and time for MBM to be 185–195 °C and 80–100 s, respectively. MBM content of 5–30% showed good uniform dispersion in bitumen mixtures, and no obvious clustering phenomenon was observed;
(5) The high-temperature stability of bitumen mixtures increased with an increased MBM content. The low-temperature performance and water stability of bitumen mixtures were at their peak with an MDM content of 20%. The road performance of DMA bitumen mixtures was best with an MDM content of 20%, which was superior to that of CMB-SBS mixtures.

## Figures and Tables

**Table 1:** Physical properties of base bitumen.

Technical Parameters	Unit	Specification	Measured Values		Test Methods
			A	B	
25 °C penetration	dmm	60–80	79.5	69.1	T0604
Softening Point	°C	≥46	48.0	48.2	T0606
15 °C ductility	cm	>100	>100	>100	T0605
60 °C dynamic viscosity	Pa·s	≥180	211	265	T0620

**Table 2:** Technical index of SBS.

Technical Parameters	Unit	Measured Values
Elongation at break	%	756
Volatiles (mass fraction)	%	0.07
Tensile strength	MPa	16.1
Relative density	—	1.045
S/B ratio	—	30/70
Appearance	—	white solid

**Table 3:** Technical indicators of PE.

Technical Parameters	Unit	Measured Values	Test Methods
15 °C density	g/cm^3^	0.92	ASTM D1505 [31]
Melt mass flow rate	g/10 min	2	ASTM D1238 [32]
Yield strength	MPa	10	ISO 527 [33]
Breaking strength	MPa	16	ISO 527 [33]
Elongation	%	780	ISO 527 [33]

**Table 4:** Technical indicators of the solubilizer.

Technical Parameters	Unit	Measured Values	Test Methods
20 °C density	g/cm^3^	0.92	ASTM D1505 [31]
20 °C flashpoint	g/10 min	200	ASTM D93 [36]
100 °C dynamic viscosity	mm^2^/s	15	ASTM D445 [37]
Condensation point	°C	−5	GB/T510 [38]

**Table 5:** CMB-SBS-modified bitumen technical performance.

Technical Parameters	Unit	Specification	Measured Values	Test Methods
25 °C penetration	dmm	60–80	55.3	T0604
Softening Point	°C	≥46	79.1	T0606
5 °C ductility	cm	>100	31.2	T0605
60 °C dynamic viscosity	Pa·s	≥180	11235	T0620
25 °C elastic recovery rate	%	≥75	92.1	T0662

**Table 6:** Technical indexes of coarse aggregate.

Technical Parameters	Unit	Specification	5–10 mm	3–5 mm
Apparent relative density	—	≥2.60	2.907	2.882
Water absorption	%	≤1.0	0.6	0.8
Crushing value	%	≤0.8	0.2	0.2

**Table 7:** Technical indexes of fine aggregate.

Technical Parameters	Unit	Specification	0–3 mm
Apparent relative density	—	≥2.50	2.770
Water absorption	%	≤12	2.6
Sand equivalent	%	≥65	72

**Table 8:** Technical indexes of mineral powder.

Technical Parameters	Unit	Specification	Measured Values
Apparent relative density	g/cm^3^	≥2.50	2.71
Moisture content	%	≤1.0	0.3
Hydrophilic coefficient	—	≤1.0	0.6
Plasticity index	%	≤4.0	3.1

**Table 9:** Capillary viscometer parameters.

Model	Diameter (mm)	Calibration Factor, 40 kPa Vacuum (Pa·s/s)			Viscosity Range (Pa·s)
		Tube B	Tube C	Tube D	
100	0.5	3.445	1.721	1.148	60–1280
800R	4.0	185.28	92.64	61.73	3800–580,000

**Table 10:** MBM in the bitumen mixture dispersion test program.

Number	Variable	Dosing (%)	Mixing Temperature (°C)	Mixing Time (s)
1	Different content	5	190	90
2		10		
3		15		
4		20		
5		25		
6		30		
7	Different content /mixing temperature	20	170	90
8			190	
9			210	
10		30	170	
11			190	
12			210	
13	Different content /mixing time	20	190	60
14				90
15				120
16		30		60
17				90
18				120

**Table 11:** Screw extrusion granulator main parameters.

Number	Model	Unit	Technical Parameters
1	Screw diameter	mm	92
2	Screw speed	r/min	65
3	Main machine power	Kw	5.5

**Table 12:** Proportion of modifier composition in MBM.

Category	SBS	PE	Solubilizer	Base Bitumen
Ratio	1	0.5	0.8	1

**Table 13:** Types of modified bitumen.

Type	0%	5%	10%	15%	20%	25%	30%
A	DMA-0	DMA-5	DMA-10	DMA-15	DMA-20	DMA-25	DMA-30
B	DMB-0	DMB-5	DMB-10	DMB-15	DMB-20	DMB-25	DMB-30

**Table 14:** Element ratio of A/B.

Elements	C	H	S	N	O
A/B Ratios	0.999	0.971	0.953	1.891	1.107

**Table 15:** Ratio of area at each peak to the sum of areas at 699 cm^−1^, 966 cm^−1^, 1377 cm^−1^, 1450 cm^−1^, 1600 cm^−1^, 2915 cm^−1^, and 2850 cm^−1^.

Type	Admixture	1377 cm^−1^	1455 cm^−1^	1600 cm^−1^	2850 cm^−1^	2919 cm^−1^
DMA	5%	6.5%	25.2%	8.9%	14.3%	43.4%
	10%	6.3%	25.0%	9.0%	14.3%	43.5%
	15%	6.3%	24.5%	9.1%	13.7%	44.1%
	20%	6.3%	24.7%	9.2%	13.3%	44.1%
	25%	6.3%	24.5%	9.2%	13.5%	43.6%
	30%	6.3%	24.6%	9.4%	13.5%	43.6%
DMB	5%	6.2%	26.7%	4.5%	14.4%	46.3%
	10%	6.4%	25.7%	4.8%	14.6%	46.3%
	15%	6.3%	26.1%	5.0%	14.2%	46.1%
	20%	6.3%	25.7%	5.2%	14.2%	16.2%
	25%	6.3%	26.5%	5.4%	14.2%	45.3%
	30%	6.7%	26.3%	5.5%	13.5%	45.2%

**Table 16:** Experimental data on the physical properties of modified bitumen.

MBM (%)	Softening Point (°C)	Penetration (dmm)	Ductility (cm)
0	47.7, 48.2, 48.1	79.0, 80.1, 79.4	0.2, 0.3, 0.4
5	52.6, 52.9, 52.6	61.0, 61.2, 61.1	26.5, 27.1, 26.8
10	60.9, 61.6, 60.8	57.3, 58.5, 58.2	29.4, 29.0, 29.2
15	77.6, 78.3, 78.4	50.5, 49.7, 50.1	38.8, 39.6, 39.2
20	86.8, 87.5, 87.0	47.2, 48.4, 47.5	40.8, 41.5, 41.3
25	88.2, 88.9, 89.0	43.1, 43.9, 43.8	36.8, 37.3, 37.5
30	92.0, 92.8, 92.1	41.6, 42.7, 42.3	39.0, 39.7, 39.5

**Table 17:** Mineral grade composition of CDP-10 bitumen mixture.

Grading Range	Percentage of Mass Passing Through the Sieve (%)								
	13.2	9.5	4.75	2.36	1.18	0.6	0.3	0.15	0.075
Gradation limit	100	100	50	32	25	16	12	10	7
Lower limit of gradation	100	85	25	18	10	7	6	5	4
Synthetic grade	100	95.5	30.4	25.9	20.4	13.0	8.5	6.3	4.9

**Table 18:** Marshall test results.

Detection Indicators	Oil/Stone Ratio (%)	Maximum Theoretical Relative Density	Void Ratio (%)	Saturation (%)	Stability (kN)	Flow Value
Test results	4.8	2.284	12.7	42.1	9.1	3.2
Technical requirements	—	—	≥10	25–45	≥8.0	1.5–4

**Table 19:** Results of the Schellenberg leakage test.

Bitumen Mixture	Specimen Number	Mix Weight (g)	Adhesive Weight (g)	Rate of Loss by Seepage (%)	Average Leakage Rate (%)	Test Temperature (°C)
CDP-10	1	1003.1	1.1	0.11	0.12	185
	2	1001.5	1.2	0.12		
	3	1002.9	1.2	0.12		

**Table 20:** Flyaway test results.

Type of Bitumen Mix	Specimen Number	Weight Before Grinding (g)	Weight After Grinding (g)	Wear Rate (%)	Average Wear Rate (%)	Dispersal Conditions
CDP-10	1	1169.2	1079.2	7.7	8.0	20 °C, 20 h. 30 r/min, 300 revolutions
	2	1173.1	1076.9	8.2		
	3	1171.4	1070.7	8.6		
	4	1172.6	1084.7	7.5		

**Table 21:** Changes in mix quality before and after the test.

Admixture	Mass Change				
	Specimen Number	$M_{c}$ (g)	$M_{s}$ (g)	$R_{MS}$ (%)	Average (%)
Base	1	60.3	58.4	3.2%	3.2%
	2	60.0	58.2	3.0%	
	3	60.1	58.1	3.3%	
5%	1	60.1	0.8	1.3%	1.6%
	2	60.2	1.1	1.8%	
	3	60.1	1.0	1.7%	
10%	1	60.1	0.6	1.0%	1.0%
	2	60.0	0.5	0.8%	
	3	60.0	0.7	1.2%	
15%	1	60.0	0.5	0.8%	0.8%
	2	60.1	0.5	0.8%	
	3	60.2	0.4	0.7%	
20%	1	60.1	0.2	0.3%	0.5%
	2	60.1	0.4	0.7%	
	3	60.0	0.3	0.5%	
25%	1	60.2	0.3	0.5%	0.4%
	2	60.0	0.2	0.3%	
	3	60.1	0.3	0.5%	
30%	1	60.0	0.2	0.3%	0.2%
	2	60.2	0.1	0.2%	
	3	60.1	0.1	0.2%	

## Data Availability

The original contributions presented in this study are included in the article. Further inquiries can be directed to the corresponding authors.
